# Virtual Reality Attention Tasks in Attention Deficit Hyperactivity Disorder: I. Behavioural Responses

**DOI:** 10.1111/ejn.70691

**Published:** 2026-09-15

**Authors:** Fjorda Kazazi, Peter Howell

**Affiliations:** ^1^ Division of Psychology and Language Sciences University College London London UK

**Keywords:** ADHD, attention, behavioural study, executive function, stuttering, virtual reality

## Abstract

Differences in executive functioning associated with working memory and different components of attention including sustained, selective, divided and attentional switching have been reported in people with attention deficit hyperactivity disorder. Traditional laboratory tasks have limited ecological validity, whereas existing virtual reality studies have focused mainly on sustained attention, with multi‐attention‐form assessment remaining relatively unexplored. Sixty‐four participants (34 people with self‐reported attention deficit hyperactivity traits and 30 neurotypical participants) were included. Participants were tested for stuttering, attention deficit hyperactivity disorder traits and phonological working memory and then completed 10 virtual reality tasks that measured all four forms of attention. The self‐reported attention deficit hyperactivity disorder group showed significantly lower attention scores, higher stuttering scores and lower phonological working memory scores. Behavioural results from the virtual reality tasks revealed that this group differed in sustained attention. Lower accuracy, higher omission error rates and reduced sensitivity occurred in this group in the traditionally formatted task based on the continuous performance test (Task 2). Logistic regression showed that all measures, except for sensitivity in Task 2, significantly predicted group membership. Results from the questionnaires indicated that overall, all participants felt present and immersed in the tasks. Findings suggest that attentional differences are form‐specific and mostly observed under sustained attention tasks. Virtual reality can be used as an ecologically valid tool for detecting form‐specific attentional differences under real‐life scenarios.

## Graphical abstract

Sustained attention was the primary domain that differentiated groups, with the traditionally formatted task being the most sensitive to attentional differences. Virtual reality paradigms may provide ecologically valid tools for form‐specific attentional assessment.

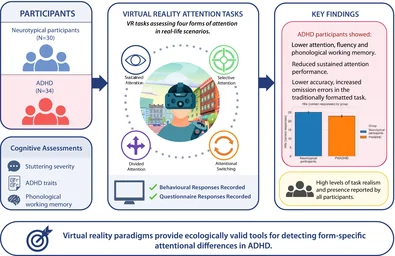

**Abbreviations**
ADHD: attention deficit hyperactivity disorder
ASRS: Adult ADHD Self‐Report Scale
AUC: area under the curve
AWADHD: adults with ADHD
CPT: Continuous Performance Test
CWADHD: children with ADHD
DSM‐5: Diagnostic and Statistical Manual of Mental Disorders 5th edition
EEG: electroencephalography
EF: executive function
fMRI: functional magnetic resonance imaging
PWADHD: people with ADHD
PWM: phonological working memory
RT: reaction time
SART: Sustained Attention to Response Task
SSI‐3: Stuttering Severity Instrument Third Edition
TFT: Traditionally Formatted Task
TMT: Trail Making Test
UNWR: Universal Nonword Repetition Test
VR: virtual reality
WM: working memory

## 1 Introduction

Executive functions (EFs) are mainly localised in the frontal cortex and involve cognitive processes related to planning, problem‐solving and attentional regulation (Miyake and Friedman 2012). These processes involve working memory (WM), inhibitory control and attentional regulation, which are important in children with attention deficit hyperactivity disorder (CWADHD; Geurts et al. 2004; Willcutt et al. 2005). Phonological working memory (PWM) differences occur in CWADHD (Martinussen et al. 2005; Rapport et al. 2008) and adults with ADHD (AWADHD; Marchetta et al. 2008). Fluency variations in CWADHD (Lee et al. 2017) and adolescents and adults with ADHD (Engelhardt et al. 2010) also occur, particularly when attentional, or speech planning, demands increase.

Attention is a core component of EF, and different forms of attention happen in everyday life. The four main forms of attention are sustained, selective, divided and switched. Examples of paradigms that assess these forms are CPT (Continuous Performance Test; Conners 2000), TFT (Traditionally Formatted Task), SART (Sustained Attention to Response Task) and visual vigilance for sustained attention; the ‘invisible gorilla’ paradigm for selective attention; TEA‐Ch (Test of Everyday Attention for Children) for divided attention; and TMT (Trail Making Tests) for attentional switching (Table 1). However, such tasks do not reflect real‐life environments. Furthermore, traditional studies in CWADHD report conflicting results for the same attention type such as for divided attention (Elosúa et al. 2017; Karatekin 2004; Table 2) and attentional switching (Elosúa et al. 2017; Pennington and Ozonoff 1996; Perugini et al. 2000; Willcutt et al. 2005). Virtual reality (VR) technology could enhance ecological validity by replicating real‐life scenarios. Current VR tasks in ADHD have focused mainly on sustained attention, especially CPT in a virtual classroom (Areces et al. 2016; Bioulac et al. 2012; Parsons et al. 2007; Pollak et al. 2009; Selaskowski et al. 2023; Wiebe et al. 2023). Other VR work has assessed CWADHD in tasks involving everyday chores (Oh et al. 2024; Salmi et al. 2024; Table 3). To our knowledge, no study has assessed people with ADHD (PWADHD) in VR across all four forms of attention.

**TABLE 1:** Four forms of attention within executive functioning.

Attention form	Definition	Typical tasks	Present study VR tasks
Sustained	The ability to stay alert and remain vigilant (Lindsay 2020)	CPT, TFT, SART, visual vigilance	Tasks 1–3
Selective	The ability to focus only on relevant stimuli while ignoring distractors (Pashler et al. 2001)	Visual search/gorilla paradigms	Tasks 4–6
Divided	The ability to process two or more tasks simultaneously	TEA‐Ch Sky Search/Score	Tasks 7, 10
Switched	The ability to alternately direct focus between several tasks (Pashler et al. 2001)	TMT/concept shifting	Tasks 8, 9

Abbreviations: CPT stands for Continuous Performance Test, SART stands for Sustained Attention to Response Task, TEA‐Ch is the Test of Everyday Attention for Children, TFT is the Traditionally Formatted Task, and TMT is the Trail Making Test.

**TABLE 2:** Summary of studies assessing PWADHD in different attention tasks including methods, statistical approaches, findings and limitations of the tasks.

Study	Attention task	Groups	Method	Statistics	Findings	Task limitations
Elosúa et al. 2017	Attentional switching visual Divided attention (verbal + motor)	26 CWADHD, 29 neurotypical participants	Trail Making (numbers/letters alternating) Digit recall at own span + box‐crossing, single then dual (mu index)	Independent *t*‐test	Slower RT in ADHD (alternating between numbers and letters); CWADHD showed no dual‐task difference and scored higher on the mu index than controls.	Limited ecological validity
Grossman et al. 2015	Selective visual (invisible gorilla)	14 AWADHD, 18 neurotypical participants	Detect unexpected stimulus (‘gorilla’)	Independent *t*‐test, *χ* ^2^ test	ADHD detected unattended changes more	Small sample; limited ecological validity
Hwang et al. 2019	A factorial go/no‐go task	49 CWADHD, 31 neurotypical participants	TFT: respond to Spiderman (go), inhibit Green Goblin (no‐go); SART: high‐go Spiderman, low‐no‐go Green Goblin	Repeated‐measures ANCOVA	ADHD severity positively correlated with error rates in both tasks	Limited ecological validity
Karatekin 2004	Divided attention (visual RT + verbal)	25 CWADHD, 27 neurotypical participants	Simple RT + counting; simple RT + digit span	Slower button presses in ADHD in simple RT + digit span	2 × 4 mixed ANOVA	Wide age range; limited ecological validity
McAvinue et al. 2012	Sustained SART visual	25 CWADHD, 25 neurotypical participants	Respond to digits except 3	Independent *t*‐test	Higher errors and greater RT variability in ADHD	Limited ecological validity
Savage et al. 2006	Divided visual–auditory	58 children with high attention difficulties, 68 children with low attention difficulties	TEA‐Ch dual tasks (visual search + auditory counting; Sky Search & Score)	Logistic regression	Dual task/visuospatial WM factor predicted attention group membership	Limited ecological validity
Swaab‐Barneveld et al. 2000	Sustained TFT visual	52 CWADHD, 55 neurotypical participants	Yes/no responses to dot patterns (go/no‐go)	One‐way ANOVA	ADHD less accurate, more impulsive, lower vigilance	Assumes normality; limited ecological validity
Tucha et al. 2009	Sustained Visual vigilance	38 AWADHD, 38 neurotypical participants; 52 CWADHD, 52 neurotypical participants	Response to visual targets (spacebar)	MANOVA	ADHD groups performed worse; no time interaction	Assumes normality; limited ecological validity

Abbreviations: AWADHD = adults with ADHD; CPT = Continuous Performance Test; CWADHD = children with ADHD; RT = reaction time; SART = Sustained Attention to Response Task; TEA‐Ch = Test of Everyday Attention for Children; TFT = Traditionally Formatted Task.

**TABLE 3:** Summary of VR studies assessing ADHD including methods, statistical approaches, findings and limitations of the tasks.

Study	Attention task	Groups	Method	Statistics	Findings	Task limitations
Areces et al. 2016	Sustained visual–auditory (AULA Nesplora)	28 neurotypical participants, 28 children inattentive, 29 children I/H, 32 children combined	AULA classroom CPT (go/no‐go; distractors; modality; motor)	MANCOVA (age, IQ); Scheffé	Separated presentations from neurotypical participants and between presentations via omissions, commissions, RT, motor activity	Non‐probability clinical sample; larger samples needed
Bioulac et al. 2012	Sustained visual (VC CPT)	20 CWADHD, 16 neurotypical participants	AK rule CPT in VC over 5 blocks; vs. CPT‐II; time‐on‐task	Mann–Whitney; Friedman; Wilcoxon; correlations	ADHD fewer hits, more commissions; performance drop over time in VC (but not CPT); VC‐CPT correlated	Small sample; boys only; limited subtypes
Oh et al. 2024	Everyday chores + AI diagnosis (AttnKare‐D) sustained + selective	15 CWADHD, 5 neurotypical participants	VR daily tasks; AI mapped into K‐ARS‐like scores	ROC; Mann–Whitney; Pearson	AUC 0.893 (cut‐off 18.44; sens. 0.80, spec. 1.0); correlated with parent/clinician K‐ARS (weaker for inattention)	Very small *n*; unequal groups; Korea‐only; weaker inattention mapping
Parsons et al. 2007	Sustained visual (VC CPT)	10 CWADHD, 10 neurotypical participants	AXE CPT ± distractors in HMD classroom; actigraphy; vs. Conners CPT II/SWAN	ANOVA; correlations; Cohen's *d*	ADHD more omissions, commissions, body movement; more distraction impact; related to SWAN/CPT	Very small pilot; boys only; neurotypical participants, higher WISC
Pollak et al. 2009	Sustained visual (VR‐CPT)	20 CWADHD, 17 neurotypical participants	VR classroom CPT vs. same CPT without VR vs. TOVA; enjoyment ratings	Repeated‐measures ANOVA	VR‐CPT distinguished groups in RT, RT variability, omission and commission errors; effect sizes similar/larger than TOVA; VR more enjoyable	Boys only; wide age range; unmedicated sample
Salmi et al. 2024	Everyday goal‐directed/EF (EPELI) sustained + selective	72 CWADHD, 71 TD	EPELI home‐chore VR; vs. CPT; ex‐Gaussian latencies	Mixed models; correlations; SVM	ADHD faster, more irrelevant actions in EPELI; CPT slower; EPELI linked more to symptoms than CPT	SES/education imbalance; EPELI ≠ pure attention construct
Selaskowski et al. 2023	Sustained visual (VSR CPT + gaze feedback)	18 AWADHD, 18 neurotypical participants	CPT in VSR; real/sham/no gaze feedback with and without distractors	Mixed ANOVA	ADHD more omissions, slower RT, more distractor gaze/head movement; feedback did not improve performance	Single session; age/education mismatch; possible sham carry‐over
Wiebe et al. 2023	Sustained visual (VSR multimodal)	25 unmedicated AWADHD, 25 medicated AWADHD, 25 neurotypical participants	CPT in VSR + actigraphy, eye‐tracking, EEG, fNIRS, experience sampling	Mixed ANOVA	Unmedicated ADHD worse CPT, more head movement/distractor gaze; medication effect mainly on omissions; no TBR/fNIRS group effects	Modest n; medication timing not tightly controlled

Abbreviations: AXE/AK = CPT target rules (respond to X or K after A); CPT = continuous performance test; I/H = impulsive/hyperactive presentation; Real vs. sham gaze feedback = immediate vs. delayed (non‐contingent) cues; sens. = sensitivity; SES = socio‐economic status; spec. = specificity; SVM = support vector machine; TBR = EEG theta/beta ratio; TD = typically developing controls; VC = virtual classroom; VSR = virtual seminar room.

Therefore, neurotypical participants and PWADHD were assessed on 10 VR tasks to understand which attention form best differentiates the groups. The study also assessed attention, fluency and PWM of PWADHD.

### 1.1 Attention and EF

EF refers to the set of abilities that enable the creation, control, regulation and execution of actions necessary to accomplish complicated goals, particularly actions that enable us to respond to new situations (Miyake and Friedman 2012). EFs are associated with the prefrontal cortex (Fuster 2015). Different frontal lobe functioning occurs in CWADHD as well as differences in impulsive, hyperactive and inattentive behaviours that are related to EF (Geurts et al. 2004). This pattern is stable over development (Biederman et al. 2007). A meta‐analysis of children and adolescents (Willcutt et al. 2005) reported that WM and multiple EFs differed in ADHD, particularly response inhibition and planning. Furthermore, Sonuga‐Barke et al. (2002) indicated that ADHD symptoms in preschool children are mainly associated with inhibitory control, but not necessarily for all aspects of EF. However, not all CWADHD show differences in these abilities (Duff and Sulla 2014; Nigg et al. 2005; Willcutt et al. 2005). Nigg et al. (2005) reported substantial overlaps in EF performance between CWADHD and neurotypical children. Although a higher proportion of CWADHD had challenges, EFs characterised only a subset of ADHD cases, highlighting its heterogeneity. Consequently, the different EFs and the patterns in ADHD performance need to be explored further. A summary of how sustained, selective, divided and attentional switching are involved in EF in everyday life, along with the typical tasks and those explored in the study, is provided in Table 1.

### 1.2 PWM and Fluency in ADHD

WM is a limited capacity system that temporarily holds information to support cognitive processes such as learning and problem solving, with verbal storage and attentional control closely related within the system (Baddeley 2012; Cowan 2013). PWM maintains verbal information via a phonological store and subvocal rehearsal, whereas the central executive coordinates this information with attentional demands.

Meta‐analytic and empirical studies in CWADHD (Martinussen et al. 2005; Rapport et al. 2008; Willcutt et al. 2005) and AWADHD (Marchetta et al. 2008) suggest WM difficulties, including verbal/PWM. In children, large differences have been observed for visuospatial WM and the central executive. CWADHD exhibit variations in fluency, particularly during story retelling (Lee et al. 2017). Adolescents and adults with ADHD‐combined type produce more mid‐utterance repairs when sentence planning demands increase (Engelhardt et al. 2010). Nonword repetition (NWR) tasks are sensitive to phonological memory skill and can be used to measure these processes (Gathercole et al. 1994) because NWR tasks measure active phonological storage. Thus, when length and phonological complexity increase, greater cognitive‐motor demands on planning and producing speech occur in NWR (Coady and Evans 2008; Smith et al. 2010).

### 1.3 Traditional and VR Tasks Assessing ADHD

Traditional studies have assessed PWADHD in sustained, selective, divided and attentional switching with findings varying across tasks (Table 2). Although such tasks provide controlled measures of attention, they have limited ecological validity. VR offers more realistic approaches when assessing attention while maintaining experimental control. However, studies using VR have predominantly assessed sustained attention in PWADHD using virtual classroom CPTs (Table 3). A meta‐analysis compared virtual classroom CPT studies (*N* = 8) with traditional 2D CPT studies (*N* = 6) and reported that virtual classrooms distinguished CWADHD from neurotypical participants with larger omissions and reaction time (RT) effects compared to 2D CPTs (Parsons et al. 2019). VR studies involving other forms of attention are rare, and multi‐attention‐form assessment has not yet been explored.

Sustained attention has been the most extensively assessed form in ADHD using CPTs, TFT, SART and visual vigilance tasks. In traditional sustained attention tasks, both CWADHD and AWADHD make more omission and commission errors and exhibit higher RT variability compared to neurotypical participants (Hwang et al. 2019; McAvinue et al. 2012; Swaab‐Barneveld et al. 2000; Tucha et al. 2009). Similar findings occur in VR tasks in virtual classroom CPTs in which both CWADHD and AWADHD make more omissions, commission and body movements and are more distracted than neurotypical participants (Areces et al. 2016; Bioulac et al. 2012; Parsons et al. 2007; Pollak et al. 2009; Selaskowski et al. 2023; Wiebe et al. 2023). The present study extended this work (Tasks 1–3; Table 1).

Selective attention tasks have been examined less often, and some have been based on the ‘invisible gorilla’ traditional task (Simons and Chabris 1999). AWADHD outperformed neurotypical participants on counting the passes and were more likely to detect the unexpected gorilla (Grossman et al. 2015). VR studies have implemented tasks that include everyday chores (e.g., get dressed and eat breakfast). CWADHD responded faster in VR, particularly for irrelevant clicks (Salmi et al. 2024), and the AttnKare tool reliably distinguished CWADHD from neurotypical participants (Oh et al. 2024). Although these approaches require participants to selectively focus on the chores, they assess broader EF rather than systematically assessing selective attention. The present study assessed this form in VR (Tasks 4–6; Table 1).

Mixed evidence on CWADHD has been reported in traditional divided tasks (Elosúa et al. 2017; Karatekin 2004). Divided tasks of ‘Sky Search and Score’ dual tasks in TEA‐Ch have been used to assess PWM, visuospatial WM and attention in CWADHD (Savage et al. 2006). Results from regression analyses indicated that group membership was significantly predicted by a visuospatial WM factor but not by verbal WM. Karatekin (2004) concluded that CWADHD may show differences in a central executive in divided tasks rather than a difference in WM storage or rehearsal. No VR study was identified that assessed divided attention in PWADHD; therefore, the present study assessed this form in Task 7 and Task 10 (Table 1).

Attentional switching in PWADHD has generally been assessed using TMT in traditional tasks. Reviews and a meta‐analysis reported that CWADHD are often slower in TMT in the condition where they had to alternate between numbers and letters (Pennington and Ozonoff 1996; Willcutt et al. 2005). Similarly, in a TMT study, CWADHD were slower in this switching condition and showed a larger time ratio of the switching condition relative to the sequential number‐only condition (Elosúa et al. 2017). However, Perugini et al. (2000) argued that this test lacks sufficient predictive power to distinguish participants. Although no VR study was identified for attentional switching, this technology offers an opportunity to assess ADHD‐related differences under more realistic conditions. Hence, this study assessed this form of attention in Tasks 8 and 9 (Table 1).

### 1.4 The Current Study

The current study conducted a multi‐attention‐form assessment to understand whether PWADHD exhibited differences mainly in sustained attention or across other forms of attention in ecologically valid VR assessments. Here, ecological validity refers to verisimilitude (resemblance to everyday environments) instead of veridicality (whether performance relates to real‐world functioning; Chaytor and Schmitter‐Edgecombe 2003). Literature suggests that to avoid referral bias, studies may benefit by using non‐clinical populations (Levy et al. 1997), and this was adopted here.

Based on the literature that suggests that attentional differences (American Psychiatric Association 2023; Chhabildas et al. 2001), fluency variations (Engelhardt et al. 2010; Lee et al. 2017) and PWM differences (Marchetta et al. 2008; Martinussen et al. 2005) occur in CWADHD and AWADHD, H1 was that the ADHD group should differ from the neurotypical group on the six‐item WHO Adult ADHD Self‐Report Screening Scale for DSM‐5^1^ (ASRS; Ustun et al. 2017), Stuttering Severity Instrument, version 3 (SSI‐3; Riley 1994) and PWM using the Universal Nonword Repetition (UNWR test, Howell et al. 2017) scores were obtained. UNWR was used here as the stimuli apply to 20 commonly spoken languages in the United Kingdom, partially addressing the disadvantage of many NWR tasks, which are language‐specific (Masoura and Gathercole 1999). Additionally, UNWR has four sets with different syllable lengths (constructed by a specified concatenation rule) that increase demands on dynamic processing. H2 was that a lower performance would be observed in PWADHD compared to neurotypical participants in sustained attention, particularly in TFT and/or SART. Furthermore, differences appear to be task‐dependent (e.g., stronger correlations between response times and CWADHD symptoms in EPELI compared with traditional CPT; Salmi et al. 2024). Given that executive control and WM demands vary across tasks, the magnitude of group differences was expected to vary across attentional forms, with differences more evident in tasks requiring greater attentional regulation and response inhibition.

## 2 Methods

### 2.1 Participants

Participants were compensated for participating in the study and travelling expenses. Admission to the university‐approved research programme required all participants to have conversational or fluent English. Accordingly, all assessments were conducted in English. The study followed the university's research ethics procedures, which require participants to understand the study materials (participant information sheet) and give written informed consent in English before testing. This study was based on Kazazi's (2023) doctoral thesis.

Thirty neurotypical participants (mean age: 23.73, SD: 3.42, 11 females and 19 males) and thirty‐four PWADHD (mean age: 24.09, SD: 5.71, 21 females and 13 males) were included in the study. Neurotypical participants were recruited from the university's SONA participant pool, and none self‐reported ADHD or other neurodevelopmental conditions. PWADHD were recruited via an opportunity sample from online ADHD groups and forums. All PWADHD reported traits of inattention and hyperactivity in line with the DSM‐5 criteria for having a presentation of ADHD. None reported a co‐occurring diagnosis or medication use. Participants were asked about alcohol and drug use prior to starting the study. As an inclusion criterion, use of drugs or alcohol was strictly prohibited. One participant was excluded on this basis due to reported alcohol use. Sensitivity power analyses were conducted for measures that significantly differentiated PWADHD from neurotypical participants. For each comparison, minimum detectable Cohen's *d* was calculated for two‐sided independent *t*‐tests for unequal sample sizes (*α* = 0.05 and 80% power). Analyses used the pwr package (pwr.t2n.test v1.3.0) in R (v4.1.3), which implements the same analytic procedures as G*Power (Faul et al. 2007), and results are reported based on the guidelines from Lakens (2022).

### 2.2 Design

The study employed a mixed experimental design where within‐participant factors were the tasks, whereas the between‐participant factor was the participant group (neurotypical participants and PWADHD). Dependent variables included background measures (ASRS, SSI‐3 and UNWR), behavioural performance measures and post‐task questionnaire responses collected after each VR task. To control for potential order effects, the order of the tasks and assessments was counterbalanced across participants.

### 2.3 Materials

#### 2.3.1 Background Measures

The ASRS consists of six items on a Likert scale from ‘*never*’ to ‘*very often*’. Responses were scored according to standard guidelines, and the total score (range 0–25) was calculated by adding up all items of the scale. ADHD symptom severity was indicated by higher scores. SSI‐3 was applied to PWADHD to measure stuttering‐like characteristics, since fluency variations have been reported in this group, particularly when speech planning and attentional demands increase and fluency sits at the intersection of attention and PWM. SSI‐3 was calculated through participant video recordings. Measures analysed included the frequency of stuttering events (e.g., repetitions, prolongations and blocks), duration of stutters (both frequency and duration measures were scaled) and physical concomitants (e.g., jaw tension). A total score was produced by summing individual scores, from which the percentile and severity classification were derived. Higher scores indicated greater stuttering‐like characteristics typical of but not exclusive to people who stutter (PWS). In the UNWR task that assessed PWM, participants repeated verbally sets of non‐words and received one point for each item they correctly produced. As the syllable length and phonological complexity increase, UNWR measures encoding, rehearsal and speech planning demands rather than phonological storage alone (Coady and Evans 2008; see also Elsherif et al. 2021; Pelczarski and Yaruss 2016). Hence, PWM connects verbal maintenance, attention and fluency speech‐motor output. The total score (range: 0–32) represented the sum of correctly repeated items, with higher scores indicating better PWM.

#### 2.3.2 VR System and Hardware

All VR tasks were developed in the C# programming language in the Unity^2^ game engine (Unity Technologies). Assets utilised in this study were downloaded from the Unity Asset Store.^3^ DAZ3D^4^ was utilised to create human avatars and their animations, whereas other animations such as particle effects were created using Unity. Audio materials were retrieved from Freesound.^5^ In some tasks, speech was generated using the IBM Watson text‐to‐speech^6^ application programming interface (API). The VR system consisted of an HTC Vive Pro^7^ head‐mounted display integrated with a Pupil Labs^8^ eye‐tracking device add‐on and a Looxid Link dry‐electrode electroencephalography (EEG) mask placed on the VR headset, connected to an Alienware m15 Ryzen Edition R7^9^ laptop. The EEG mask had six channels (AF3, AF4, AF7, AF8, Fp1, Fp2; FPz reference) and was built to fit within the VR headset used here. The company (Looxid Labs) now provides other VR health products, and the EEG mask is no longer sold because it does not fit newer VR headsets.

#### 2.3.3 VR Task Environments

##### 2.3.3.1 Task 1—Living Room

In Task 1, participants were placed in a living room setting that contained 3D objects such as a sofa, table, TV, fireplace and kitchen area. At the start of the task, a video was played on TV with a news reporter talking about a specific topic. The fireplace had a particle effect and fire sound. Two human avatars were positioned on the left (female avatar) and right side (male avatar) of the participant. The female avatar alternated gaze between TV and the participant while commenting on the news. The male avatar held its gaze on the TV while performing eating chips animations. Other elements included kitchen sounds, such as appliance noises.

##### 2.3.3.2 Tasks 2 and 3—Lecture Hall

Tasks 2 and 3 included a virtual classroom environment in which the participant was seated among student avatars representing peers. A lecturer avatar delivered a lecture in front of the classroom while lecture images were displayed on a large screen. Surrounding student avatars produced behaviours and sounds such as coughing, sneezing and writing. A book was placed in front of the participant, and a paper plane object was included within the environment and displayed at a specific time.

##### 2.3.3.3 Tasks 4 and 5—Street

Tasks 4 and 5 were conducted in an outdoor urban environment consisting of a cityscape with buildings, streets, vehicles and pedestrian avatars. Ambient sounds included traffic noise and people talking. Animated birds, cars and a flying plane were present. The participant was placed in front of a park, with two roads located on each side of the park.

##### 2.3.3.4 Task 6—Bar

Task 6 included a virtual bar with 3D tables, chairs and ambient background music. Multiple human avatars were positioned around the user and had animations such as talking, dancing, drinking and preparing drinks. Three human avatars were placed in front of the participant and discussed a topic. An animated non‐human character (alien‐like figure) walked in front of the user at a specific time.

##### 2.3.3.5 Task 7—Street Pedestrian

Task 7 included a virtual city environment containing 3D models of buildings, roads, vehicles, traffic lights, parks and pedestrian avatars. A mobile phone object with ringtone and notification sounds was placed in front of the user during the task. Other elements included birds with animations and ambient city sounds. Movement of the vehicles was achieved using behavioural logic in Unity. Cars moved only when traffic signals were green, and a honking sound played if the participant was in the middle of the street and the car traffic signals were green.

##### 2.3.3.6 Task 8—Train Station

Task 8 environment included a virtual train station and 3D models such as trains, a ticket machine and platform elements. Ambient sounds included train announcements, doors opening and closing, movement of trains and general station noises. Human avatars were placed in the scene with corresponding movements and speech animations. One avatar was placed in front of the participant and delivered a spoken message.

##### 2.3.3.7 Task 9—Elevator

In Task 9, participants were placed inside a virtual elevator which contained interactive buttons and a mobile phone object. Background sounds included elevator movement, door opening and closing and elevator music. At the start of the task, two human avatars initiated a conversation.

##### 2.3.3.8 Task 10—Chemistry Lab

Task 10 took place in a virtual chemistry laboratory that contained equipment, a periodic table and a robot 3D model placed on a table. For chemical reactions, particle effects and their associated sounds were presented.

### 2.4 Procedure

Participants were first assessed for vision and hearing. During the vision test, they sat 1 m from the computer and, with one eye covered, they indicated the orientation of the letter E, which was reduced in size. The experimenter clicked the direction indicated by participants to record a response. To proceed with the next test, participants had to correctly identify the orientation of the stimulus with both eyes. For the hearing test, participants responded aloud when they detected tones played through Optimus Nova 626 headphones at various intensities (0–50 dB) and frequencies (0.25–8 kHz). They were unaware of the intensity and frequency of the tone and were required to detect tones at or below 20 dB across all frequencies to continue. After this step was completed, participants conducted the background measures and VR tasks. The order of the three background tasks was counterbalanced to remove order effects. For the first 10 participants, the order was ASRS, SSI‐3, all 10 VR tasks and UNWR. For the next 10 participants, the orders were UNWR, VR tasks, ASRS and SSI‐3. This was repeated across the sample. VR tasks and the order of questions in the questionnaires were also counterbalanced.

#### 2.4.1 Background Measures

Participants completed the ASRS tool, read two short paragraphs from the SSI‐3 manual while being video recorded (participants consented to being video‐recorded) and conducted the UNWR task while the experimenter wrote down the scores. In the UNWR task, participants first heard two practice non‐words through Optimus Nova 626 headphones to familiarise with the task, followed by the eight non‐words in a random order (automatically set by the program) in each UNWR set (four sets in total) played by the experimenter. After each non‐word was played, participants repeated them aloud. If the non‐word was repeated correctly, the experimenter proceeded to the next item; if not, the task ended. They were required to successfully repeat all eight non‐words to progress to the next set. To minimise the effect of accent differences, scoring was based on consonants only, following Howell et al. (2017).

#### 2.4.2 VR Tasks

**FIGURE 1:**
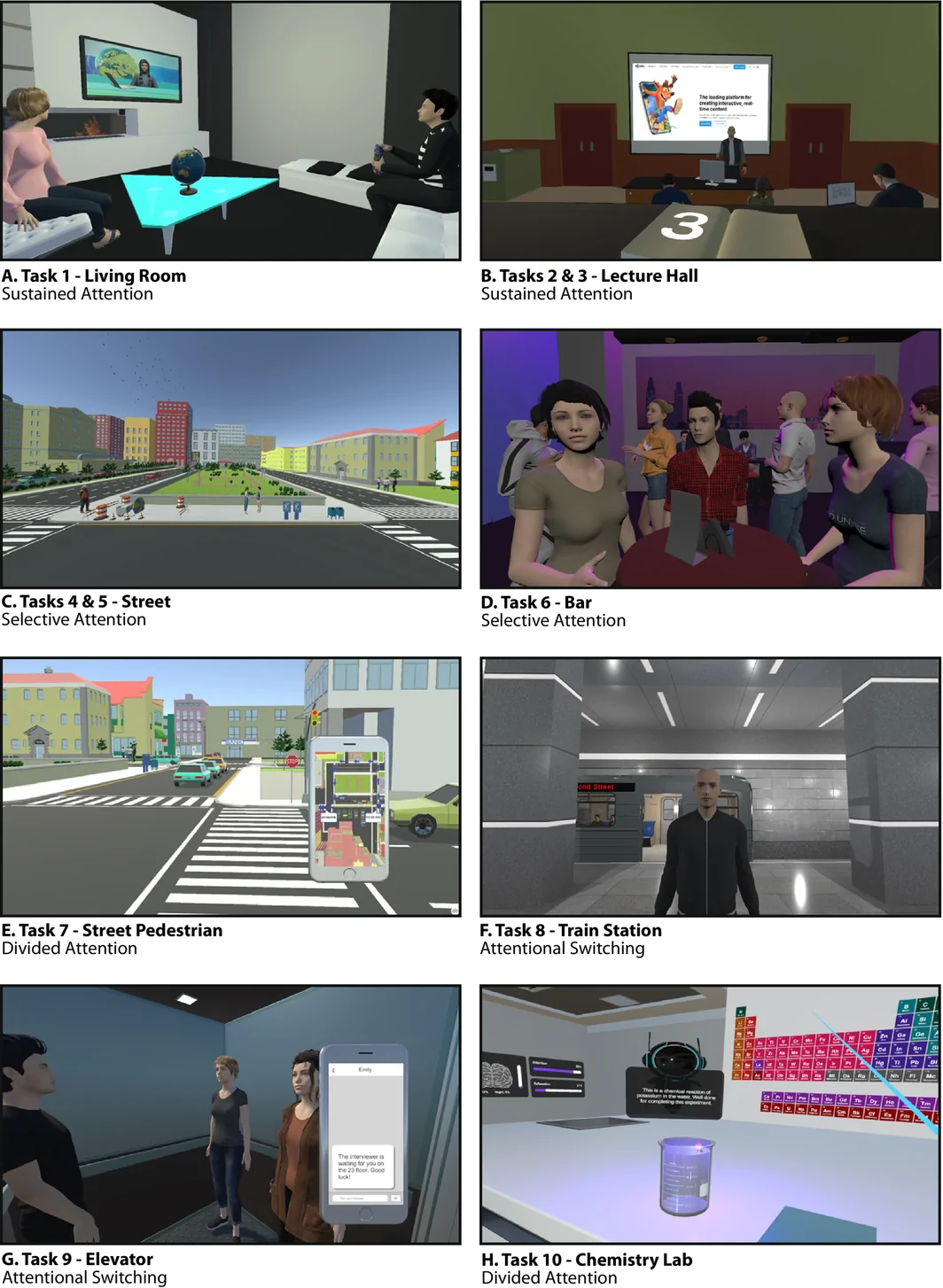
VR tasks utilised in the current study. (A) Task 1—Living room measured sustained attention; (B) Tasks 2 and 3—Lecture hall measured sustained attention (CPT/TFT and SART); (C) Tasks 4 and 5—Street measured selective attention; (D) Task 6—Bar measured selective attention; (E) Task 7—Street pedestrian assessed divided attention; (F) Task 8—Train station assessed attentional switching; (G) Task 9—Elevator measured attentional switching; and (H) Task 10—Chemistry lab measured divided attention. Tasks 2–3 and Tasks 4–5 used the same environments.

The first 10 participants completed tasks from 1 to 10; the next 10 participants completed tasks from 10 to 1 (Figure 1). Before each task, they read the instructions and were given the opportunity to ask the experimenter any questions. They were familiarised with the VR environment in advance. A calibration procedure was conducted prior to each VR task to ensure that participants were correctly positioned within VR and for accurate eye‐tracking data collection. Practice trials were included for VR tasks 2, 3, 4, 5, 7, 8 and 10 and were conducted after participants read instructions. Tasks 1 and 6 did not require a practice trial, as they involved listening only and did not require any user interaction. After each task, users completed a questionnaire related to the task and to the VR technology. Oculomotor and questionnaire data were obtained for all tasks, whereas behavioural data were obtained for all tasks except for Task 6, as this task did not involve attention components from which behavioural measures could be derived. EEG data were recorded in all 10 VR tasks. After inspection, raw and spectral were not analysed. The mask used dry forehead sensors and often lost contact when the headset was adjusted. The mask was also affected by blinks, saccades, facial and body movements and the research‐grade artefact correction (e.g., removing blinks) was not feasible since only processed data were available. However, the company sold the EEG mask mainly as a Brain Computer Interface (BCI) product, and its intended function was to use a real‐time attention index (0–1, updated at 10 Hz) to interact with the objects in the VR. Only this index was retained and used in Task 10.

##### 2.4.2.1 Task 1—Living Room

Participants sat on a sofa and were asked to pay attention to a TV news reporter while ignoring distractions from two avatars and ambient sounds. The task lasted approximately 3 min and measured visual and auditory sustained attention.

##### 2.4.2.2 Tasks 2 and 3—Lecture Hall

For both tasks, the digits 1 through 9 were presented one at a time in random order in the book in front of the participants. In total, the tasks had 225 trials, and each digit appeared 25 times. Digits were presented for 1.15 s, and the delay between digits was set to 1.15 s. In Task 2, for a correct response to be recorded, participants had to press the VR controller button only when digit 3 appeared. In Task 3, they were asked to press the button for all digits except for digit 3. Participants were also told to ignore distractions such as the lecturer, student avatars and a flying paper plane shown at a given time. The tasks lasted approximately 4.5 min each. Both tasks had a limit of 10 min. Tasks 2 and 3 were designed as CPT/TFT and SART formats to assess visual sustained attention in a lecture setting comparable to virtual classroom CPTs.

##### 2.4.2.3 Tasks 4 and 5—Street

Cars (50 in total with random colours) appeared randomly one at a time on the left or right side (25 cars on each side). Each car was displayed for 1.15 s, and the delay between the cars was 1.15 s. Participants looked towards the road where the car appeared and pressed the corresponding button (Task 4: selective visual attention) or looked and pressed the opposite road to the car (Task 5: selective visual attention) while ignoring other environmental distractions. Each task lasted approximately 3.5 min and had a 10‐min limit.

##### 2.4.2.4 Task 6—Bar

Participants had to count the number of times a target word was spoken by avatars during the conversation, whereas an alien character briefly appeared at a specific time. The task measured selective visual and auditory attention and was 1.5 min long.

##### 2.4.2.5 Task 7—Street Pedestrian

Participants memorised a map (which they could reopen as needed, shown for 3 s) to navigate from point A to point B by crossing five roads while simultaneously checking missed calls and text messages (both shown for 3 s) and responding to an incoming phone call (lasting 6 s) presented on a virtual mobile device. The task measured visual and auditory divided attention and had a 10‐min limit.

##### 2.4.2.6 Task 8—Train Station

Participants followed instructions from an avatar to catch the correct train. Trains stopped at the platform for 10 s, and the delay between trains was 2 s. The task measured visual and auditory attentional switching over a 10‐min period.

##### 2.4.2.7 Task 9—Elevator

Participants used the elevator buttons to reach the correct floor after listening to cues from avatar conversations and checking a phone prompt. The task measured visual and auditory attentional switching and was limited to 10 min.

##### 2.4.2.8 Task 10—Chemistry Lab

Participants completed three chemistry experiments under robot instruction, while interacting with laboratory equipment. The attention index was used as an interaction threshold and not as a diagnostic inclusion rule or a clinical attention cutoff. Participants interacted with the chemistry lab equipment only if this index was above 0.4. This threshold was set below Looxid's interaction demos cutoff of 0.5 so that the task could be completed by all participants. This index (averaged across the task) was saved as a value from 0 to 1 and was analysed here. The task measured visual divided attention and had a limit of 10 min. Additionally, the task score (0–100 value) was calculated by adding 20 points to the mean attention index (multiplied by 100), from which a time penalty based on the time to complete the task was subtracted (0 at the start up to 2 at the 10‐min time limit).

#### 2.4.3 Questionnaires on VR Tasks

After conducting each VR task, participants also completed, within the VR headset, a questionnaire with two questions related to the realism of VR tasks and feeling present in the task.

## 3 Results

A range of variables were analysed for each VR task for behavioural data. Data analysis excluded variables with minimal variability or floor or ceiling effects. Outliers in both background and behavioural measures were winsorized at 1.5 × IQR (extreme values were capped at 1.5 × IQR from the quartiles), and any missing data due to technical recording issues were excluded. Sample sizes for each background and behavioural measures, as well as Shapiro–Wilk tests, are included in Tables 4 and 5, respectively. For background measures, only two participants were missing in each group in SSI‐3, and missingness did not differ between groups (Fisher's exact *p* = 1.00; effective *N* = 60). For behavioural measures, missingness did not differ between groups in Task 1 (*χ* ^2^ = 0.00, *p* = 1.00; effective *N* = 20), Task 4 (Fisher's exact *p* = 0.659; effective *N* = 59) or Task 10 (Fisher's exact *p* = 0.494; effective *N* = 62). Remaining background measures and behavioural tasks, as well as questionnaires, had complete data (*N* = 64). Independent‐samples *t*‐tests or Mann–Whitney *U* tests were conducted as appropriate (Tables 6 and 7 for background and behavioural measures, respectively) alongside effect sizes (Cohen's *d*). Age and gender were compared between groups and used as covariates for background and behavioural measures in tasks with significant group differences. Count variables were modelled using generalised linear models (GLMs) to account for non‐normal distributions and overdispersion. To determine whether the ADHD group assessed in the present study based on self‐reported ADHD symptoms were comparable to individuals with a clinical ADHD diagnosis, a linear mixed model (LMM) was conducted to check whether their ASRS, SSI‐3 and UNWR scores were comparable to an independent sample assessed with the same measures that included participants with a clinical ADHD diagnosis. Two models were created. The first model looked at the effect of two fixed factors: (1) measure type ASRS, SSI‐3 or UNWR reflecting the overall between attention, fluency and PWM processes observed in PWADHD; and (2) subtype (neurotypical participants, self‐reported ADHD, clinically diagnosed ADHD); and two random factors (participant and group). The second model looked at the effect of one fixed factor: measure type (ASRS, SSI‐3 or UNWR) and the two random factors (participant and group). Therefore, only one model included subtype. No significant difference in the model fit was observed (*χ* ^2^(1) = 3.13, *p* = 0.077). Hence, the self‐reported PWADHD in this study did not differ significantly from the clinically diagnosed group, supporting the use of PWADHD data as a reasonable representation of ADHD‐related symptom profiles.

**TABLE 4:** Descriptive Statistics for ASRS, SSI‐3 and UNWR.

	Group									
	Neurotypical participants					PWADHD				
Measure	***N***	***M***	SD	Outl.	Norm.	***N***	***M***	SD	Outl.	Norm.
ASRS	30	10.38	3.08	1	< 0.001	34	16.81	2.38	1	0.003
SSI‐3	28	1.04	2.25	0	< 0.001	32	4.78	5.30	0	< 0.001
UNWR	30	21.93	4.02	0	0.497	34	17.24	5.68	0	0.750

Abbreviations: Outl. and Norm. represent outliers and normality, respectively.

**TABLE 5:** Descriptive Statistics for variables in each task.

		Groups									
		Neurotypical participants					PWADHD				
Task	Measure	***N***	***M***	SD	Outliers	Normality	***N***	***M***	SD	Outliers	Normality
1	Looked TV count	9	894.33	982.98	0	0.027	11	1685.27	713.54	0	0.132
	Mean duration TV	9	237.20	134.58	3	0.037	11	176.78	75.19	1	< 0.001
2	Hits	30	25.10	2.42	3	0.007	34	22.88	3.48	4	0.028
	Misses	30	0.77	1.33	0	< 0.001	34	2.57	2.92	3	< 0.001
	Overall accuracy (%)	30	99.85	1.38	3	0.102	34	98.76	1.88	3	0.067
	Target accuracy (%)	30	100.40	9.69	3	0.007	34	91.53	13.94	3	0.028
	Omission error rate (%)	30	3.07	5.32	0	< 0.001	34	10.29	11.66	3	< 0.001
	Overall error rate (%)	30	0.58	0.91	0	< 0.001	34	1.46	1.61	1	< 0.001
	Mean Hit RT (ms)	30	596.52	67.92	0	0.782	34	610.00	95.24	1	0.827
	SD Hit RT (ms)	30	85.85	36.31	0	0.027	34	100.79	46.93	0	0.099
	*d′* (sensitivity)	30	4.92	1.02	0	0.001	34	4.41	1.22	13	0.006
3	Hits	30	195.69	6.60	3	< 0.001	34	195.93	6.76	3	0.001
	Misses	30	4.68	6.25	3	< 0.001	34	4.66	6.14	3	< 0.001
	Overall accuracy (%)	30	96.14	3.47	3	< 0.001	34	96.00	3.59	3	0.031
	Target accuracy (%)	30	97.84	3.30	3	< 0.001	34	97.97	3.38	3	0.001
	Omission error rate (%)	30	2.34	3.12	3	< 0.001	34	2.33	3.07	3	< 0.001
	Overall error rate (%)	30	4.01	3.29	3	< 0.001	34	4.25	3.28	3	0.003
	Mean Hit RT (ms)	29	536.78	79.84	0	0.693	34	535.63	88.72	1	0.936
	SD Hit RT (ms)	29	127.15	47.15	1	0.082	34	127.15	42.48	1	0.016
	*d′* (sensitivity)	30	3.55	1.46	2	0.038	34	3.60	1.42	11	0.018
4	Overall accuracy (%)	27	95.84	8.93	2	< 0.001	32	90.88	11.93	4	< 0.001
	Mean Hit RT (ms)	27	782.18	162.11	3	0.009	32	726.04	151.39	1	0.007
	SD Hit RT (ms)	27	146.99	40.69	1	0.597	31	154.58	45.70	2	0.536
5	Overall accuracy (%)	30	95.63	8.66	1	< 0.001	34	88.79	14.50	8	< 0.001
	Mean Hit RT (ms)	30	852.76	154.99	1	0.026	34	818.31	179.26	1	0.019
	SD Hit RT (ms)	30	172.65	35.06	1	0.645	34	167.61	43.69	3	0.043
7	Map presses (count)	30	15.78	13.45	4	< 0.001	34	17.22	10.24	3	< 0.001
	Map presses per second	30	0.04	0.03	3	0.008	34	0.04	0.02	1	0.008
	CT (ms)	30	398,831.27	132,546.62	0	0.042	34	399,610.41	93,449.74	0	0.073
8	CT (ms)	30	68,921.75	47,305.40	0	0.034	34	79,267.90	66,287.44	1	0.003
9	Phone presses (count)	30	4.22	3.00	1	0.023	34	5.26	3.10	2*	0.266
	Phone presses per second	30	0.47	0.42	0	0.017	34	0.71	0.56	3*	0.007
	CT (ms)	30	12,428.92	8883.65	3	< 0.001	34	11,193.34	8837.65	4*	< 0.001
10	Attention (avg)	30	0.590	0.071	0	0.010	32	0.636	0.051	0	< 0.001
	Task score	30	78.33	7.21	0	0.010	32	83.13	5.10	0	< 0.001
	CT (ms)	30	168,707.48	57,755.05	1	0.157	32	144,883.97	53,663.46	1	0.003

Abbreviations: Mean Hit RT stands for mean reaction time for hit responses, SD Hit RT is the standard deviation or variability of reaction time on hit responses, and CT stands for completion time.

**TABLE 6:** Between‐group significance (*t*‐test and Mann–Whitney *U*).

Measure	Independent *t*‐test	Cohen's *d*	Mann–Whitney *U*	Interpretation
ASRS	*t*(62) = −9.399, *p* < 0.001	2.35	*U* = 25.5, *p* < 0.001	PWADHD > Neurotypical
SSI‐3	*t*(58) = −3.475, *p* = 0.001	0.899	*U* = 282.0, *p* = 0.004	PWADHD > Neurotypical
UNWR	*t*(62) = 3.773, *p* < 0.001	0.945	*U* = 767.0, *p* < 0.001	Neurotypical > PWADHD

*Note:* Cohen's *d* represents effect size (0.20 = small, 0.50 = medium, 0.80 = large).

**TABLE 7:** Between‐group significance (*t*‐test and Mann–Whitney *U*).

Task	Measure	Independent *t*‐test	Cohen's *d*	Mann–Whitney *U*	Direction
1	Looked TV count	*t*(18) = −2.085, *p* = 0.052	0.94	*U* = 23.0, *p* = 0.048*	PWADHD > NP
	Mean TV duration	*t*(18) = 1.271, *p* = 0.220	0.57	*U* = 53.5, *p* = 0.790	NP > PWADHD
2	Hits	*t*(62) = 2.918, *p* = 0.005*	0.73	*U* = 679.5, *p* = 0.019*	NP > PWADHD
	Misses	*t*(62) = −3.117, *p* = 0.003*	0.78	*U* = 344.5, *p* = 0.013*	PWADHD > NP
	Overall accuracy (%)	*t*(62) = 2.616, *p* = 0.011*	0.66	*U* = 670.5, *p* = 0.029*	NP > PWADHD
	Target accuracy (%)	*t*(62) = 2.918, *p* = 0.005*	0.73	*U* = 679.5, *p* = 0.019*	NP > PWADHD
	Omission error rate (%)	*t*(62) = −3.117, *p* = 0.003*	0.78	*U* = 344.5, *p* = 0.013*	PWADHD > NP
	Overall error rate (%)	*t*(62) = −2.665, *p* = 0.010*	0.67	*U* = 358.0, *p* = 0.031*	PWADHD > NP
	Mean Hit RT (ms)	*t*(62) = −0.644, *p* = 0.522	0.16	*U* = 475.0, *p* = 0.643	PWADHD > NP
	SD Hit RT (ms)	*t*(62) = −1.410, *p* = 0.164	0.35	*U* = 424.0, *p* = 0.250	PWADHD > NP
	*d′* (sensitivity)	*t*(62) = 1.830, *p* = 0.072	0.46	*U* = 655.5, *p* = 0.047*	NP > PWADHD
3	Hits	*t*(62) = −0.145, *p* = 0.885	0.04	*U* = 505.5, *p* = 0.957	PWADHD > NP
	Misses	*t*(62) = 0.014, *p* = 0.989	0.00	*U* = 505.0, *p* = 0.950	NP > PWADHD
	Overall accuracy (%)	*t*(62) = 0.151, *p* = 0.881	0.04	*U* = 534.0, *p* = 0.751	NP > PWADHD
	Target accuracy (%)	*t*(62) = −0.145, *p* = 0.885	0.04	*U* = 505.5, *p* = 0.957	PWADHD > NP
	Omission error rate (%)	*t*(62) = 0.014, *p* = 0.989	0.00	*U* = 505.0, *p* = 0.950	NP > PWADHD
	Overall error rate (%)	*t*(62) = −0.284, *p* = 0.777	0.07	*U* = 479.5, *p* = 0.686	PWADHD > NP
	Mean Hit RT (ms)	*t*(61) = 0.054, *p* = 0.957	0.01	*U* = 502.0, *p* = 0.907	NP > PWADHD
	SD Hit RT (ms)	*t*(61) = −0.001, *p* = 1.00	0.00	*U* = 489.0, *p* = 0.962	NP = PWADHD
	*d′* (sensitivity)	*t*(62) = −0.141, *p* = 0.888	0.04	*U* = 518.0, *p* = 0.919	PWADHD > NP
4	Overall accuracy (%)	*t*(57) = 1.780, *p* = 0.081	0.47	*U* = 534.0, *p* = 0.075	NP > PWADHD
	Mean Hit RT (ms)	*t*(57) = 1.375, *p* = 0.175	0.36	*U* = 527.5, *p* = 0.148	NP > PWADHD
	SD Hit RT (ms)	*t*(56) = −0.663, *p* = 0.510	0.18	*U* = 380.5, *p* = 0.559	PWADHD > NP
5	Overall accuracy (%)	*t*(62) = 2.254, *p* = 0.028*	0.57	*U* = 609.0, *p* = 0.140	NP > PWADHD
	Mean Hit RT (ms)	*t*(62) = 0.817, *p* = 0.417	0.21	*U* = 594.0, *p* = 0.261	NP > PWADHD
	SD Hit RT (ms)	*t*(62) = 0.505, *p* = 0.615	0.13	*U* = 564.5, *p* = 0.468	NP > PWADHD
7	Map presses (count)	*t*(62) = −0.48, *p* = 0.631	0.12	*U* = 393.0, *p* = 0.116	PWADHD > NP
	Map presses per second	*t*(62) = −0.56, *p* = 0.578	0.14	*U* = 440.5, *p* = 0.353	NP = PWADHD
	CT (ms)	*t*(62) = −0.03, *p* = 0.978	0.01	*U* = 487.0, *p* = 0.762	PWADHD > NP
8	CT (ms)	*t*(62) = −0.710, *p* = 0.480	0.18	*U* = 487.0, *p* = 0.762	PWADHD > NP
9	Phone presses (count)	*t*(62) = −1.37, *p* = 0.176	0.34	*U* = 392.0, *p* = 0.112	PWADHD > NP
	Phone presses per second	*t*(62) = −1.95, *p* = 0.056	0.49	*U* = 382.0, *p* = 0.086	PWADHD > NP
	CT (ms)	*t*(62) = 0.56, *p* = 0.580	0.14	*U* = 556.0, *p* = 0.540	NP > PWADHD
10	Attention (avg)	*t*(60) = −2.953, *p* = 0.005*	0.75	*U* = 299.0, *p* = 0.011*	PWADHD > NP
	Task score	*t*(60) = −3.036, *p* = 0.004*	0.77	*U* = 287.5, *p* = 0.007*	PWADHD > NP
	CT (ms)	*t*(60) = 1.684, *p* = 0.097	0.43	*U* = 603.0, *p* = 0.084	NP > PWADHD

*Note:* Cohen's *d* represents effect size (0.20 = small, 0.50 = medium, 0.80 = large).

Abbreviations: CT stands for completion time; NP stands for neurotypical participants.

* *p* < 0.05.

### 3.1 Background Measures Data Analysis

**FIGURE 2:**
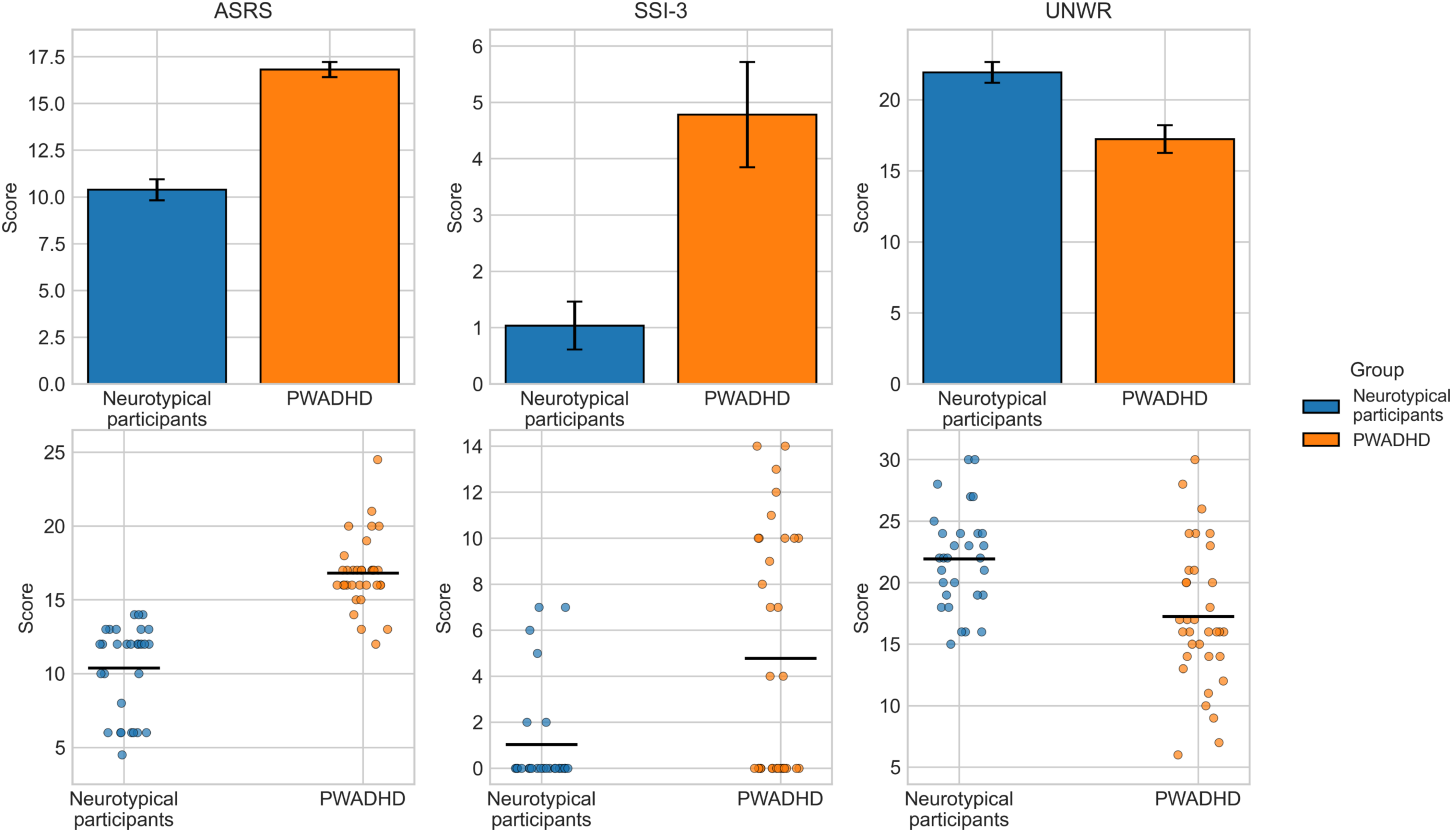
Top row representing bar graphs visualising group differences on ASRS, SSI‐3 and UNWR scores. Error bars represent ±SEM. The bottom row shows individual participant scores on ASRS, SSI‐3 and UNWR, with horizontal lines indicating group means.

As mentioned, SSI‐3 data were missing for two participants in each group, and there were no missing data in other measures. Two outliers were identified in ASRS. Normality tests indicated that ASRS and SSI‐3 were non‐normal in both groups, whereas UNWR was normally distributed, so both independent‐samples *t*‐tests and Mann–Whitney *U* tests were conducted where appropriate. Independent‐samples *t*‐tests revealed that PWADHD scored significantly higher than neurotypical participants on the ASRS, *t*(62) = −9.399, *p* < 0.001, *d* = 2.35, and SSI‐3, *t*(58) = −3.475, *p* = 0.001, *d* = 0.899. In UNWR, the neurotypical group demonstrated significantly higher scores than PWADHD, *t*(62) = 3.77, *p* < 0.001, *d* = 0.945. Mann–Whitney *U* tests confirmed the same pattern of results for ASRS (*U* = 25.5, *p* < 0.001) and SSI‐3 (*U* = 282.0, *p* = 0.004). Tables 4 and 6 and Figure 2 provide more detailed information. The effects observed were larger than the minimum detectable effects from the sensitivity power analysis (*d* = 0.71 for ASRS and UNWR; *d* = 0.74 for SSI‐3), suggesting that the study was sufficiently powered for the background‐measure comparisons reported here. Age did not differ significantly between groups in ASRS (*t* = −0.31, *p* = 0.761), SSI‐3 (*t* = −0.64, *p* = 0.522) and UNWR (*t* = −0.31, *p* = 0.761). Likewise, gender did not differ significantly between groups in ASRS (*χ* ^2^(1) = 3.08, *p* = 0.080), SSI‐3 (*χ* ^2^(1) = 3.28, *p* = 0.070) and UNWR (*χ* ^2^(1) = 3.08, *p* = 0.080). After controlling for age and gender using ANCOVAs, groups remained significant for all measures (all *p* < 0.05).

### 3.2 Behavioural Data Analysis

#### 3.2.1 Task 1

**FIGURE 3:**
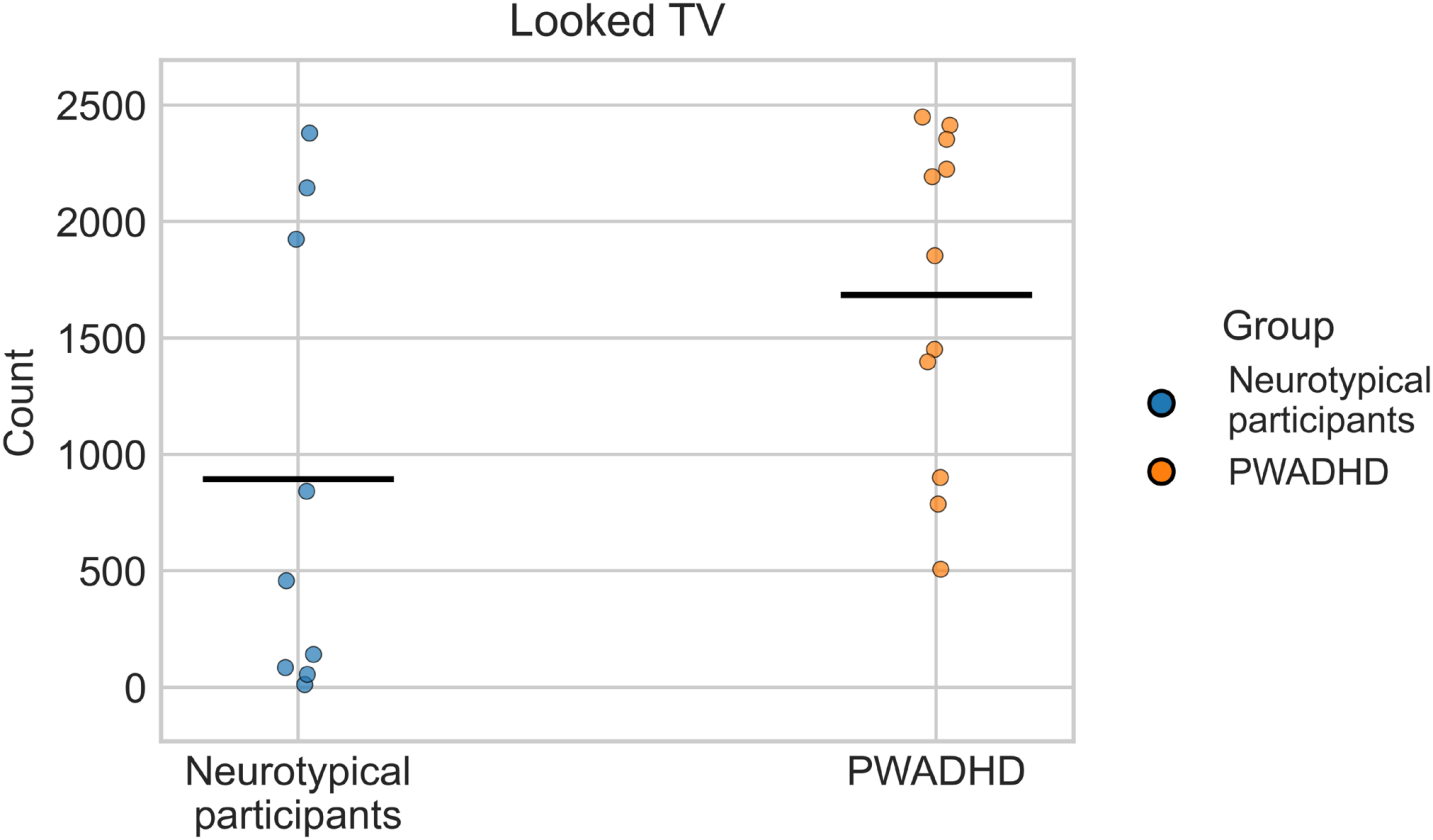
Dot plots visualising group differences in attention frequency (looked TV count) in Task 1. Points represent individual participants. Horizontal lines indicate group means.

**FIGURE 4:**
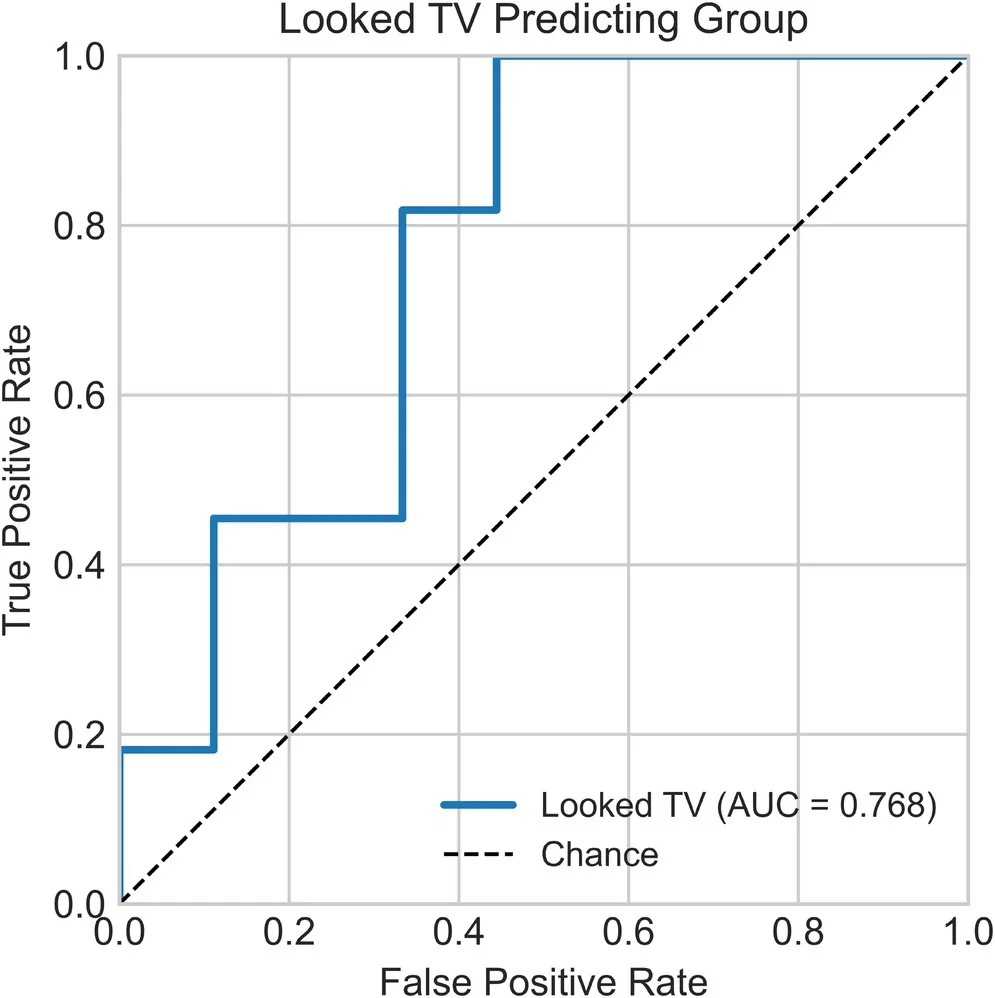
Receiver operating characteristic (ROC) curve for Task 1 looked TV count predicting group membership (neurotypical participants and PWADHD). The diagonal line indicates chance performance (AUC = 0.50).

In Task 1, participants focused on the target TV, which included an auditory–visual stimulus while ignoring distractions. Twenty participants were included in the analysis due to technical issues (Table 5). Shapiro–Wilk tests indicated non‐normality for attention frequency (looked TV count) in the neurotypical group (*p* = 0.027) and for mean TV duration in both groups (*p* < 0.05). An independent‐samples *t*‐test showed a trend for higher attention frequency in PWADHD (*t*(18) = −2.09, *p* = 0.052, *d* = 0.94), with a significant difference in this variable as indicated from Mann–Whitney *U* test (*U* = 23.0, *p* = 0.048). Mean TV duration was lower in PWADHD but did not differ significantly (*t*(18) = 1.27, *p* = 0.220; *U* = 53.5, *p* = 0.790, *d* = 0.57). Table 7 provides more information. With 20 participants, the design was sensitive to between‐group effects of Cohen's *d* = 1.33. The effect for attention frequency (*d* = 0.94) was large, but below this threshold, indicating limited power. Age and gender did not differ significantly between groups (*t* = −1.89, *p* = 0.077; *χ* ^2^(1) < 0.01, *p* = 1.00). GLM for attention frequency confirmed a significant group effect (IRR = 3.98, *p* = 0.005), with PWADHD looking at the TV approximately four times more frequently than neurotypical participants (Figure 3). Logistic regression (Figure 4) showed that looked TV count had moderate discriminative accuracy for group membership (AUC = 0.77, 95% CI [0.53, 0.97]; model *p* = 0.043) with classification accuracy of 70% (sensitivity was 72.7%, specificity was 66.7%). Although no significant differences were observed in the duration of attention to the target, PWADHD showed a higher frequency in attention shifts to the target.

#### 3.2.2 Tasks 2 and 3

**FIGURE 5:**
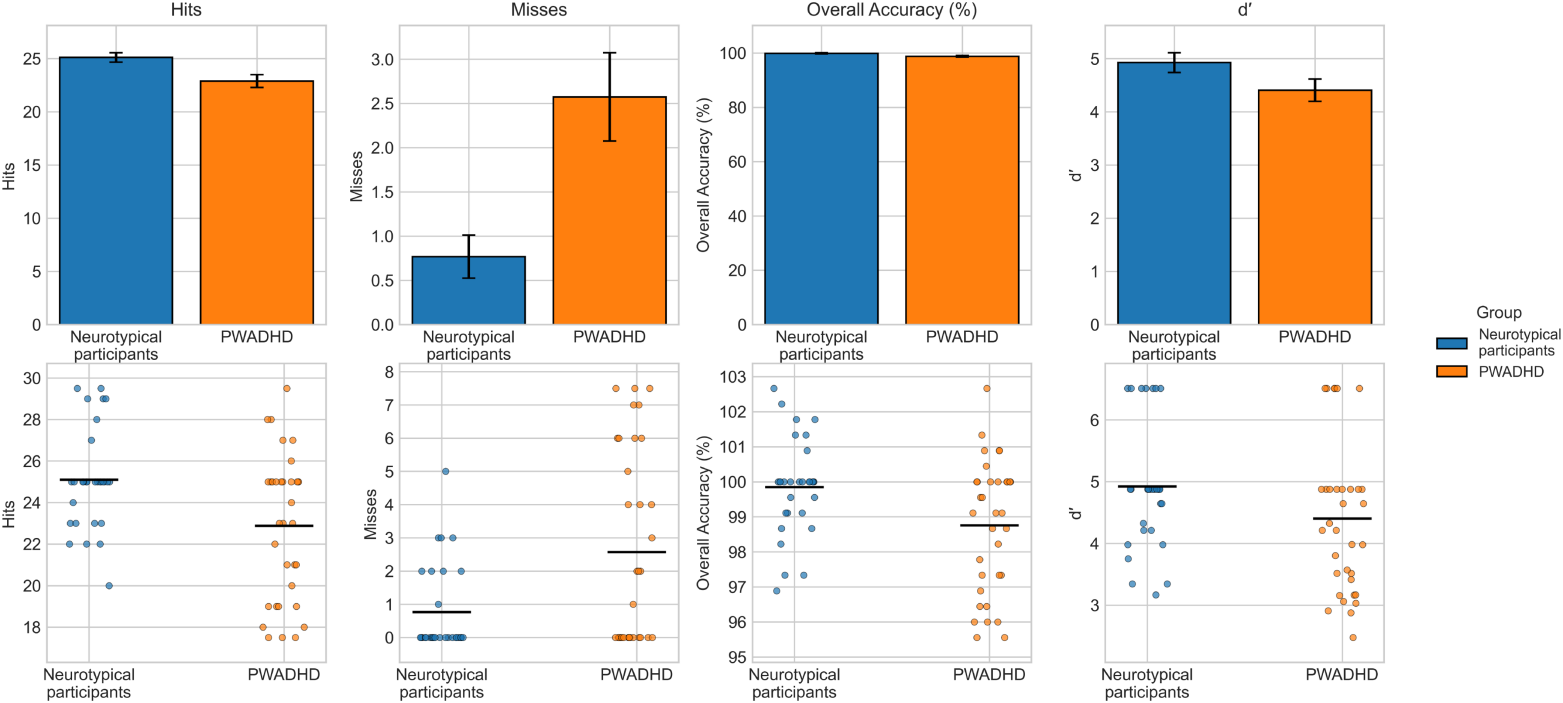
Bar graphs (top row) and participant data points (bottom row) visualising group differences for hits (correct responses), misses (omission errors), overall accuracy % and *d′* (sensitivity) in Task 2. Error bars in the top row represent ±SEM, and horizontal lines in the bottom row represent group means.

**FIGURE 6:**
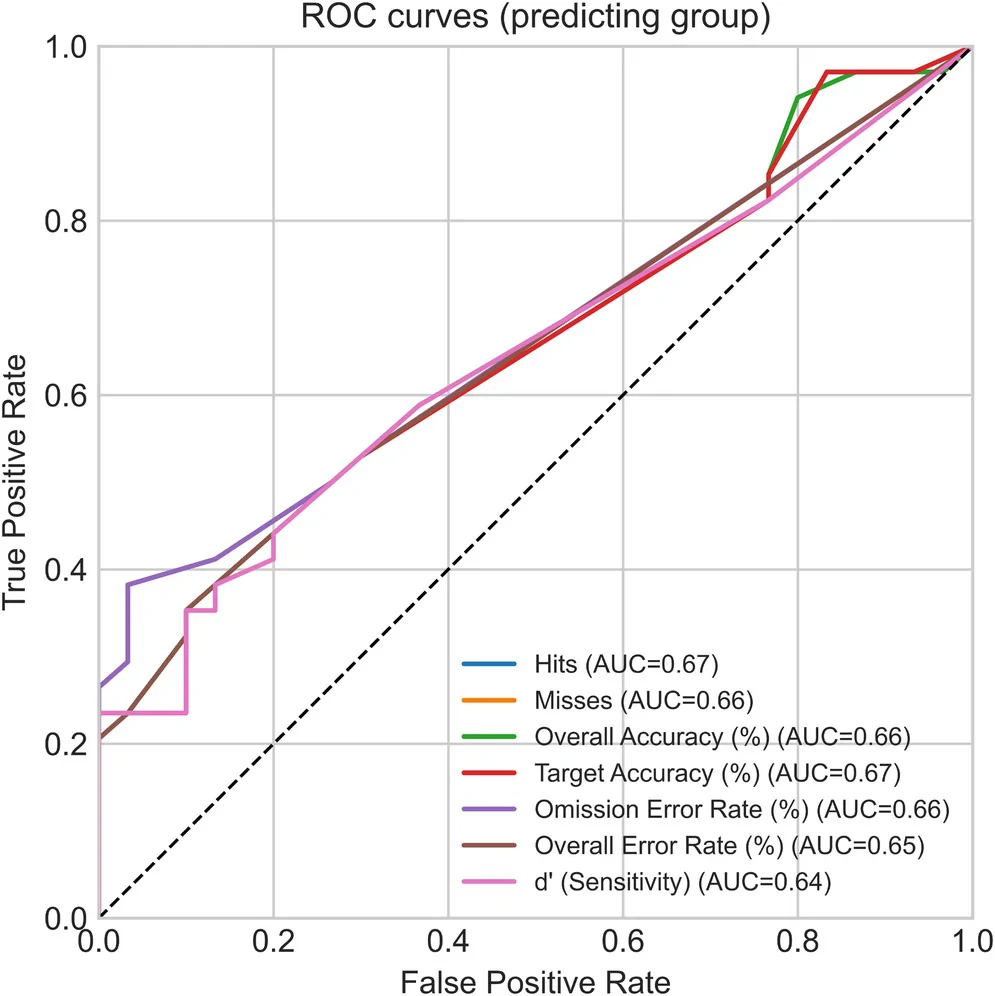
Receiver operating characteristic (ROC) curve for Task 2 hits (correct responses), misses (omission errors), overall accuracy %, target accuracy %, omission error rate %, overall error rate % and *d′* (sensitivity) predicting group membership (neurotypical participants and PWADHD). The diagonal line indicates chance performance (AUC = 0.50).

There were no missing data in Task 2 (TFT) and 1 missing data in Mean Hit RT and variability of Hit RT (SD Hit RT) in Task 3 (SART; Table 5). In Task 2, hits, misses, target accuracy, omission error rate, overall error rate, and *d′* were non‐normal, as indicated by Shapiro–Wilk. Normality was met for overall accuracy and Mean Hit RT in both groups, and SD Hit RT in PWADHD (*p* > 0.05). In Task 3, most variables were non‐normal (*p* < 0.05) except Mean Hit RT in both groups and SD Hit RT in the neurotypical group (*p* > 0.05). PWADHD showed lower Hits and higher Misses in TFT, with *t*‐tests indicating significant group differences (Hits: *t*(62) = 2.92, *p* = 0.005, *d* = 0.73; Misses: *t*(62) = −3.12, *p* = 0.003, *d* = 0.78) and corresponding Mann–Whitney *U* tests (Hits: *U* = 679.5, *p* = 0.019; Misses: *U* = 344.5, *p* = 0.013). PWADHD also exhibited lower overall accuracy (*t*(62) = 2.616, *p* = 0.011), target accuracy, higher omission error rate and overall error rates (all significant; *p* < 0.05), whereas *d′* showed a trend towards lower sensitivity in PWADHD (*t*(62) = 1.83, *p* = 0.072, *d* = 0.46, *U* = 655.5, *p* = 0.047; Figure 5). Mean Hit RT did not differ significantly between groups. Between‐group comparisons in SART using *t*‐tests and Mann–Whitney *U* tests showed no significant differences in any behavioural measure (all *p* > 0.05). See Table 7 for more information. Sensitivity power analysis for Task 2 indicated that the design was sensitive to between‐group effects of Cohen's *d* = 0.71. Observed effects for Hits (*d* = 0.73) and Misses (*d* = 0.78) exceeded this threshold. In Task 2, no significant differences between groups were observed with respect to age (*t* = −0.31, *p* = 0.761) and gender (*χ* ^2^(1) = 3.08, *p* = 0.080). ANCOVAs controlling for age and gender confirmed group effects for Hits, Misses, overall accuracy, target accuracy, omission error rate and overall error rate (all *p* < 0.05), whereas *d′* remained non‐significant (*p* = 0.055). Logistic regression for Task 2 (Figure 6) showed that Misses and omission error rate had the strongest statistical association for group membership (*p* = 0.002; AUC = 0.66, 95% CI [0.54, 0.78]). Hits, target accuracy (%), overall accuracy (%) and overall error rate (%) were also significant (*p* = 0.04–0.010; AUC 0.65–0.67, 95% CIs approximately [0.52, 0.79]). Classification accuracy was approximately 61% (sensitivity was 50%–59%, specificity was 63%–73%), indicating moderate discriminative accuracy. There was no significant predictive group power for *d′* (*p* = 0.068; AUC = 0.64, 95% CI [0.52, 0.77]; accuracy 54.7%), and it had poor specificity (23.3%) and thus did not effectively discriminate between groups.

#### 3.2.3 Task 4 and 5

**FIGURE 7:**
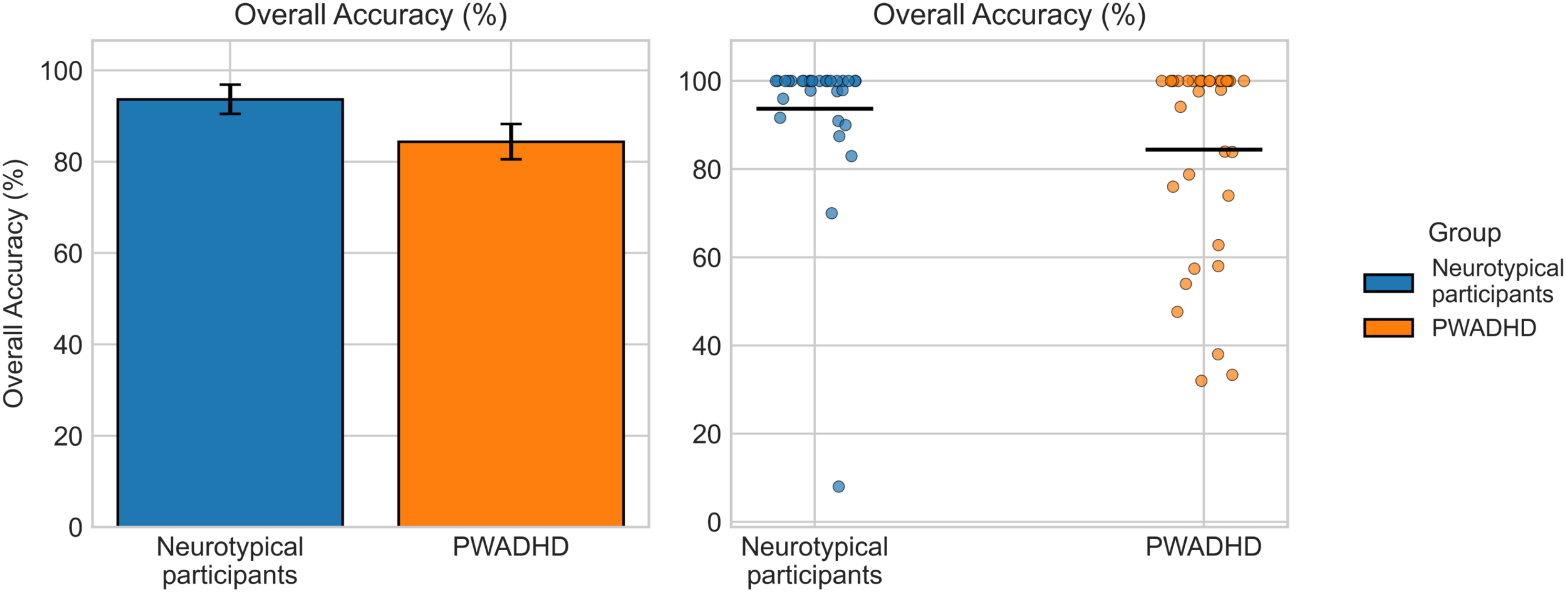
Bar graphs (left plot) and participant data points (right plot) visualising group differences for overall accuracy (%) in Task 5. Error bars represent ±SEM, and horizontal lines represent group means.

**FIGURE 8:**
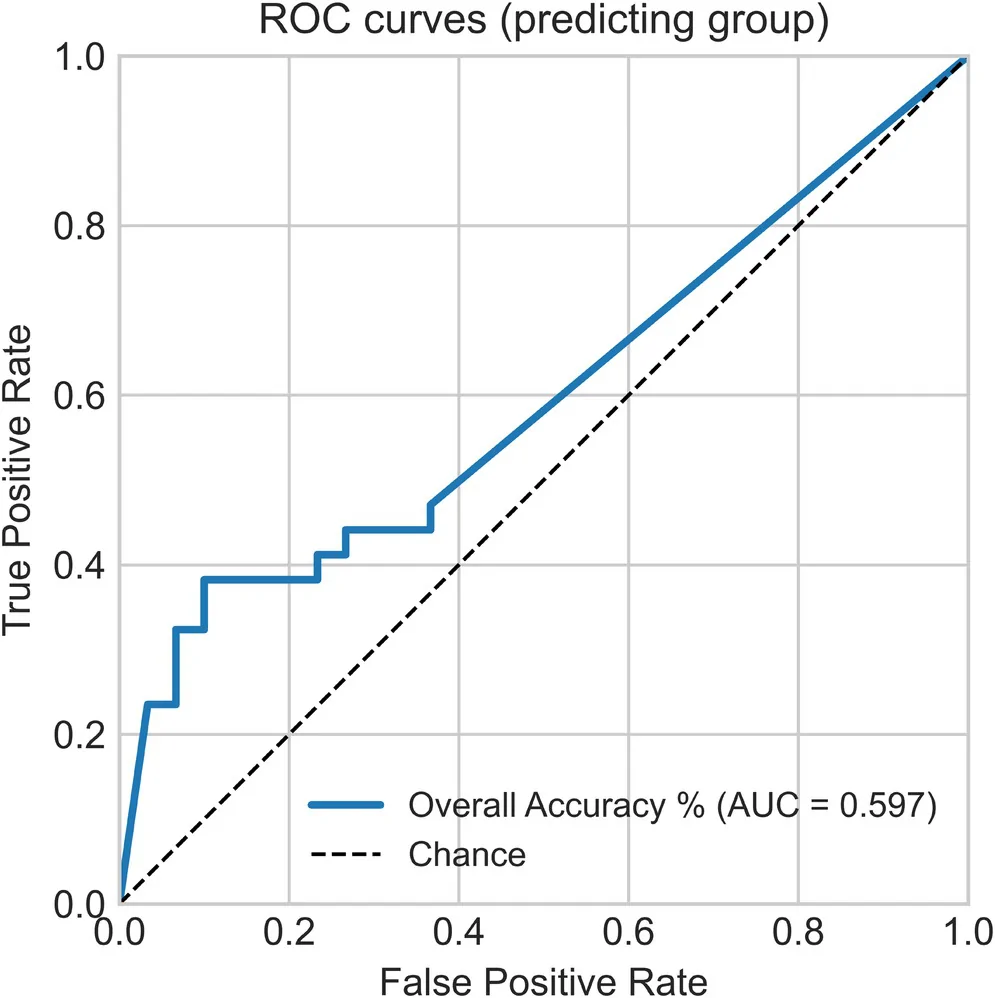
Receiver operating characteristic (ROC) curve for Task 5 overall accuracy predicting group membership (neurotypical participants and PWADHD). The diagonal line indicates chance performance (AUC = 0.50).

Fifty‐nine participants were included in Task 4, and no participants were missing in Task 5 (Table 5). Shapiro–Wilk tests indicated non‐normality for overall accuracy and Mean Hit RT in both groups (*p* < 0.05), whereas SD Hit RT was normal (*p* > 0.05) in the neurotypical group in both tasks and PWADHD (Task 4). As shown in Table 7, *t*‐tests and Mann–Whitney *U* tests revealed no significant differences between groups for any variable in Task 4, although a nonsignificant trend suggested lower overall accuracy in PWADHD. In contrast, in Task 5, a significant group difference was observed in overall accuracy, as shown in Figure 7 (*t*(62) = 2.254, *p* = 0.028, *d* = 0.565), and sensitivity analysis showed that the design was sensitive to between‐group effects of Cohen's *d* = 0.71. The observed accuracy effect (*d* = 0.57) was medium but below this threshold, indicating limited power. In Task 5, age and gender did not differ significantly between groups (*t* = −0.31, *p* = 0.761; *χ* ^2^(1) = 3.08, *p* = 0.080). Overall accuracy remained significant after controlling for age and gender using ANCOVA (*p* = 0.037). Logistic regression showed that this measure significantly predicted group membership but with poor to modest discrimination (*p* = 0.024; AUC = 0.60, 95% CI [0.48, 0.72]). Accuracy was 57.8%, sensitivity was 41.2%, and specificity was 76.7% (Figure 8).

#### 3.2.4 Task 7

No participant data were missing (Table 5). Shapiro–Wilk tests indicated non‐normality for map presses (count; *p* < 0.001) in both groups, map presses per second in both groups (*p* = 0.008) and for completion time (CT) in the neurotypical group (*p* = 0.042). No significant group differences were observed in all variables as assessed by the independent‐samples *t*‐tests (all *p* > 0.05) and Mann–Whitney *U* test (Table 7). GLM confirmed no significant group effect for the count variable (IRR = 1.18, *p* = 0.538).

#### 3.2.5 Task 8

There were no missing data as shown in Table 5, and Shapiro–Wilk tests indicated non‐normality for CT in both groups (*p* < 0.05). PWADHD had longer CTs than the neurotypical group, but this difference was not significant based on the independent‐samples *t*‐test (*t*(62) = −0.71, *p* = 0.480, *d* = 0.18) and the Mann–Whitney *U* test (*U* = 487.0, *p* = 0.762; Table 7).

#### 3.2.6 Task 9

There were no missing data (Table 5). CT and phone presses per second were non‐normal in both groups, as indicated by Shapiro–Wilk tests. Normality was met for phone presses only in PWADHD. Independent‐samples *t*‐tests and Mann–Whitney *U* tests indicated that groups did not differ for any variables (Table 7). Consistent with the parametric and non‐parametric analyses, GLM showed no significant group effect (*p* > 0.05).

#### 3.2.7 Task 10

**FIGURE 9:**
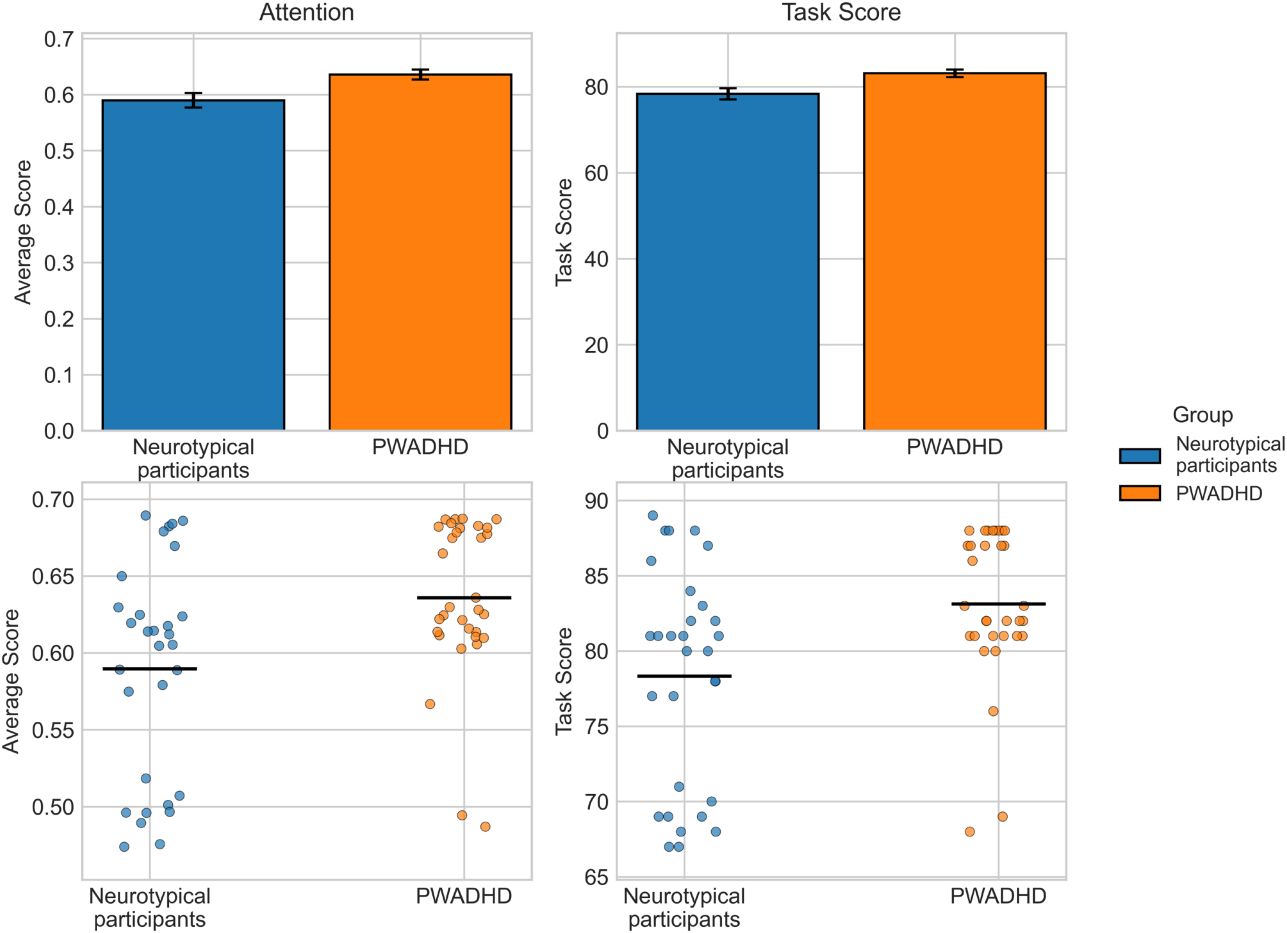
Bar graphs (top row) visualising group differences for attention (average) and score in Task 10. Scatter plot (bottom row) visualising participant data points for the same variables. Error bars represent ±SEM, and horizontal lines represent group means.

There were no missing data in the neurotypical group, and two participants from the ADHD group were missing behavioural data due to technical recording issues (Table 5). Shapiro–Wilk tests indicated non‐normality for attention average and score in both groups (*p* < 0.05) and for CT in PWADHD only (*p* = 0.003). Attention average and task scores were significantly higher in PWADHD (*t*(60) = −2.95, *p* = 0.005, *d* = 0.75; *U* = 299.0, *p* = 0.011; *t*(60) = −3.04, *p* = 0.004, *d* = 0.77; *U* = 287.5, *p* = 0.007) compared to neurotypical participants (Table 7 and Figure 9). CT was lower in PWADHD but did not differ significantly between groups. The design was sensitive to between‐group effects of Cohen's *d* = 0.72, as indicated by sensitivity analysis. Observed effects for attention average (*d* = 0.75) and task score (*d* = 0.77) exceeded this threshold. Age and gender did not differ significantly between groups (*t* = −0.25, *p* = 0.806; *χ* ^2^(1) = 3.17, *p* = 0.075). After controlling for age and gender using ANCOVA, attention average and task score remained significant (*p* = 0.013; *p* = 0.011).

### 3.3 Questionnaire Data Analysis

Across all 10 VR tasks, participants reported high levels of presence (mean ratings: 3.58–4.11), all significantly above the neutral midpoint (all *t* > 4.8, all *p* < 0.001). Task realism was also generally high (mean ratings: 3.08–4.08) with means above the neutral point (all *t* > 3.0, all *p* ≤ 0.003), except for Task 3 (*t*(63) = 0.57, *p* = 0.568). Overall, 62%–86% of participants agreed or strongly agreed that they felt present, and 41%–86% rated the tasks as realistic. Presence and realism ratings were positively correlated across tasks (Spearman *ρ* = 0.42–0.64, all *p* < 0.001), indicating that higher perceived presence was associated with greater perceived task realism.

## 4 Discussion

### 4.1 Summary

The present study compared PWADHD and neurotypical participants within VR scenarios that resemble real‐life across sustained, selective, divided and attentional switching. It also investigated whether attention, fluency and PWM differences occur in PWADHD. Consistent with H1 and the literature, PWADHD demonstrated lower attention, fluency and PWM compared to neurotypical participants, as indicated by ASRS, SSI‐3 and PWM scores. Results are consistent with the literature suggesting differences in attention, speech and PWM in CWADHD and AWADHD (American Psychiatric Association 2023; Chhabildas et al. 2001; Engelhardt et al. 2010; Lee et al. 2017; Marchetta et al. 2008; Martinussen et al. 2005). Exploratory analyses were conducted to examine whether these measures intercorrelated. Spearman correlations within groups and after controlling for group did not reach statistical significance, although the association between SSI‐3 and UNWR in the PWADHD group was moderate. Findings suggest that although background differences in PWADHD are observed within a broader pattern, these measures do not necessarily vary together at the individual level. The same pattern was observed in a related study assessing a larger sample of neurotypical participants, PWADHD and PWS on the same measures through network models (NMs; Kazazi and Howell 2026). In our NM study, we found that despite overlapping characteristics of attention, fluency and PWM, the groups showed partially different cognitive architectures. NMs of PWADHD and neurotypical participants were similar, whereas both differed from the NM of PWS. Taken together, findings may indicate that the relationship between attention, fluency and PWM may differ depending on the broader pattern of relationships. Thus, ADHD‐related differences may reflect a more complex organisation of these processes.

In line with H2, and consistent with past research, group differences were most evident in sustained attention. In Task 1 (sustained visual), PWADHD looked at the target (TV) more frequently but for shorter durations, indicating lower and unstable attention. This is in line with past research that suggests that PWADHD exhibit a less stable attentional behaviour and a greater rate in gaze and movement directed to distractions (Parsons et al. 2007; Selaskowski et al. 2023; Wiebe et al. 2023), indicating difficulties in maintaining focus. This pattern may also reflect increased demands on executive control mechanisms which are responsible for maintaining task goals and attention regulation. Executive processes support both attentional regulation and PWM maintenance, and such findings may reflect broader executive control differences relevant for verbal WM demands in PWADHD. However, due to the reduced sample size, the results are interpreted as exploratory. The clearest support for H2 are the results obtained in the TFT (CPT go/no‐go paradigm; Task 2), in which PWADHD exhibited lower hit rates, more omission errors and reduced accuracy, with significant predictive power, aligning more closely with the virtual classroom CPT in which CWADHD (Areces et al. 2016; Bioulac et al. 2012; Parsons et al. 2007; Pollak et al. 2009) as well as AWADHD (Selaskowski et al. 2023; Wiebe et al. 2023) exhibit more omissions, reduced hit performance and poorer sustained vigilance relative to neurotypical groups. Findings may indicate that in paradigms with infrequent targets, PWADHD may face difficulties detecting rare targets, and such differences become more apparent in ecologically valid VR approaches that include distractors, as seen in VR classroom studies. Such results are also in line with meta‐analytic evidence, which indicates that VR classroom CPTs compared to traditional 2D CPTs may better differentiate the ADHD group from neurotypical participants, often showing larger omissions in this group (Parsons et al. 2019). Findings also converge with traditional TFT/CPT paradigms in both children and adults with ADHD (Hwang et al. 2019; Swaab‐Barneveld et al. 2000; Tucha et al. 2009) as well as evidence showing difficulties in inhibitory control in CWADHD (Nigg 2001; Sonuga‐Barke et al. 2002; Willcutt et al. 2005). Findings may also reflect difficulties in maintaining focus on the task and regulating attention over time in the presence of distractions. These processes rely on central executive resources within WM and are consistent with the link between attentional regulation and PWM, as both require maintaining attention and resisting interference. In contrast with studies suggesting slower RTs in CWADHD and AWADHD (Pollak et al. 2009; Selaskowski et al. 2023) but in line with other studies in CWADHD (Bioulac et al. 2012; Parsons et al. 2007), Mean Hit RTs in Task 2 did not differ significantly between groups. This suggests that PWADHD show lapses in attention during rare‐target detection rather than exhibiting slower responses. Although *d′* did not predict group membership, it differed between groups, indicating that hit/miss indices were more informative than a sensitivity metric in this paradigm. In contrast to previous SART findings in CWADHD in traditional tasks (Hwang et al. 2019; McAvinue et al. 2012) as well as in AULA VR tasks (Areces et al. 2016), no group differences were observed in the SART. This discrepancy may be sample‐related since results are largely from child clinical samples whereas the current study assessed self‐reported adults. Hence, developmental stage and clinical status may influence the findings. Supplementary logistic regressions were conducted and interpreted as exploratory rather than as diagnostic tests. Findings showed that Tasks 1 and 2 had the strongest discriminative accuracy for group membership (Task 1 looked TV count: AUC = 0.77, accuracy = 70.0%; Task 2 omission errors: AUC = 0.66, accuracy = 60.9%). However, findings in Task 1 are interpreted more cautiously due to the reduced sample size. Additional exploratory regression analyses were conducted to predict Task 1 (look frequency at the TV, mean look duration) and Task 2 (hits, misses, overall accuracy, *d′*) from ASRS, SSI‐3 and UNWR with and without group. Task 1, showed that performance was related to higher ASRS scores (frequent and shorter looks at the TV). After controlling for group, only mean duration remained related to ASRS (*p* = 0.003). Task 2 related to ADHD group membership rather than the background measures. Such differences in Task 2 may reflect that this task taps into sustained attention as well as inhibitory demands in which ASRS may not map directly. Although overlapping background measures of PWM and fluency can coexist in PWADHD as in neurotypical participants (Kazazi and Howell 2026), real‐time differences may emerge during sustained attention tasks rather than as stuttering‐like characteristics and PWM mechanism. This interpretation is also in line with the broader VR finding that ADHD differences are most evident during the sustained attention form, supporting the view that ADHD is heterogeneous and context‐dependent (Nigg et al. 2005; Willcutt et al. 2005; Salmi et al. 2024).

Findings in the selective attention tasks were mixed. Although Task 4 showed no differences, PWADHD had a lower performance compared to neurotypical participants in Task 5 in overall accuracy with limited discriminative power. Such findings are consistent with the literature that differences in PWADHD are evident when tasks require greater executive control and participants need to focus on the task while suppressing automatic responses and ignoring distractions. Group differences observed suggest that PWADHD may have more difficulty with tasks that require higher levels of cognitive control. Results on overall accuracy in Task 5 cannot be interpreted as difficulties in simple response inhibition since participants indicated an alternative response rather than withholding their response. Instead, PWADHD may have had difficulties in managing competing responses and maintaining task goals under higher cognitive demands similar to VR studies in CWADHD (Salmi et al. 2024). However, the moderate effect size (*d* = 0.57) and classification accuracy (57.8%) indicate that the design had limited power and was not accurate enough to reliably differentiate groups. Hence, findings are interpreted as exploratory. Grossman et al. (2015) reported that AWADHD were more likely to detect the unexpected stimuli in the invisible gorilla paradigm, suggesting reduced inattentional blindness rather than attentional difficulties. Such findings highlight that attentional performance in ADHD is strongly influenced by task demands. Children and adults with ADHD are more prone to direct their attention to irrelevant information (Salmi et al. 2024; Selaskowski et al. 2023; Wiebe et al. 2023) while also showing difficulties in maintaining goal‐directed behaviour under higher executive control demands (Salmi et al. 2024). Task 6 replicated the invisible gorilla paradigm and will be further assessed by oculomotor measures in Part II.

No group differences were observed in the divided attention street navigation task (Task 7), in contrast with divided task differences reported in traditional paradigms such as TEA‐Ch (Savage et al. 2006). Findings may be explained by the structure of the task. Participants were able to reopen the map, manage navigation and phone events based on their pace, which may have allowed them to use strategies that are less available in more controlled divided tasks. The self‐paced structure may have reduced demands on resources of central executive and may have allowed participants to externally support task completion rather than relying on internal processes of WM. In addition, the measures assessed may have been relatively broad (e.g., CT and map use), and subsequently, smaller difficulties in managing simultaneous visual and auditory information may not have been detected. Notably, in the VR chemistry lab (Task 10), PWADHD exhibited higher engagement and better performance. However, after controlling attention index and CT, outcomes were no longer predicted by groups, suggesting that the results depended on how PWADHD engaged in the task rather than to a separate group effect. Additionally, VR neurofeedback and reward training approaches have been shown to improve ADHD symptoms in children and adolescents, often by reducing the levels of inattention or impulsiveness, and can improve inhibitory control (Cho et al. 2004; Fang et al. 2024). Task 10 introduced a neurofeedback (NF)‐like demand, but since participants conducted the task in a single session, the findings cannot be interpreted as those achieved from NF training tasks. However, the design is closer to a training‐style task rather than an assessment of ADHD, as it requires participants to stay engaged in the task to complete it. Hence, the results may be explained by the design as well as being a meaningful goal‐oriented VR task. This is consistent with VR findings in which differences in CWADHD depended on goal‐directed activity and response style (e.g., faster or more frequent actions in EPELI; Salmi et al. 2024) rather than on lower accuracy alone. Findings support the literature suggesting that differences in PWADHD are not necessarily evident only by an inability to pay attention but also require regulating attention based on the task demands and motivation. Furthermore, results are in line with training studies that show that meaningful VR contexts can enhance engagement and performance in both CWADHD and AWADHD (Cunha et al. 2023; Shema‐Shiratzky et al. 2018).

Across attentional switching tasks (Tasks 8 and 9), differences were small with similar RTs observed between groups in contrast with traditional studies such as the TMT that report slower RTs in CWADHD (e.g., Elosúa et al. 2017). This contrast may be explained by the design of classic TMT paradigms, which measure attentional switching and performance also depends on speed and visuomotor demands as well as developmental stage. Additionally, VR scenarios distribute attentional demands across more natural and familiar cues. Hence, classic attentional switching paradigms may not generalise to ecologically valid environments or may only emerge under specific cognitive loads.

Overall, high levels of presence and realism across tasks were reported by participants, indicating immersion in the tasks. Importantly, group differences did not reflect overall disengagement in PWADHD (e.g., Task 10). This is in line with arguments that suggest that executive performance in ADHD is sensitive to environmental structure and motivational salience. VR paradigms allowed assessment of attentional differences in stimulating real‐life scenarios while maintaining experimental control. Such paradigms address concerns regarding the artificiality of traditional tasks that assess attention. The results showed that group differences are not observed in all forms of attention when attentional demands are measured in meaningful scenarios. Such results were observed in VR classroom CPTs, everyday‐chore paradigms and multimodal assessments, in which differences in ADHD are form‐specific and, in particular, under sustained attention tasks. In the present study, the more pronounced group differences were found for sustained attention, whereas differences in selective, divided and attentional switching were less pronounced across the 10 VR tasks.

### 4.2 Theoretical and Clinical Implications

The main theoretical implication of this study is that EF differences in PWADHD are form‐specific. In particular, sustained attention under low‐go demands appears to be the most sensitive to group differences, whereas selective, divided and attentional switching may not be significantly affected under ecologically valid conditions. Findings are in line with research that suggests a heterogeneous view in PWADHD and emphasises the importance of the task and the environment in assessing cognitive function. Additionally, the role of task structure and motivational salience is also important. In line with the literature, meaningful contexts (e.g., Task 10) may improve engagement and performance. Executive outcomes in ADHD are sensitive to environmental and motivational factors (Salmi et al. 2024). VR technology can be utilised as a tool to assess ADHD. However, performance may depend on several factors such as familiarity, individual interaction styles and comfort with VR headsets. Additionally, VR scenarios offer a verisimilitude approach to ecological validity, and results may reflect their maximal performance rather than real‐world functioning (Chaytor and Schmitter‐Edgecombe 2003; Toplak et al. 2013).

The main clinical implication is that assessing PWADHD in different forms of attention may help identify where attentional difficulties are more likely to emerge. In the present sample, sustained attention paradigms under low‐go demands appear to be most informative. However, the moderate discriminative accuracy in this task suggests that clinical assessments should also consider task format, medication use, ADHD presentations, co‐occurring neurodevelopmental conditions, symptom ratings and clinical history alongside VR behavioural measures. Hence, a multimodal approach may increase discriminative accuracy for group membership. Additionally, tasks implemented to train attention in ADHD may benefit from VR technology, which offers engaging environments (Cunha et al. 2023; Shema‐Shiratzky et al. 2018), while also taking into consideration that performance may still differ from typical everyday functioning. In a systematic review and meta‐analysis study of randomised controlled trials (RCTs), Zheng et al. (2025) concluded that immersive VR interventions can reduce ADHD‐related symptoms in CWADHD and may offer greater ecological validity and engagement compared to non‐immersive VR tasks. Cao et al. (2026) systematic review reported that VR interventions used either with or without medication improved ADHD symptoms in children and adolescents. Longer interventions of ≥ 8 weeks were associated with better and more sustained results. In a related study (Coleman et al. 2019), performance of 15 children with ADHD‐related symptoms exhibited lower omission errors, hit variability, RT and RT variability after Cogmed WM training. It was suggested that psychometrically validated VR assessments may provide additional information beyond parent or teacher reports. Shema‐Shiratzky et al. (2018) trained 14 CWADHD in a divided task VR paradigm (walking on a treadmill while clearing virtual obstacles). After training, parent‐rated social and psychosomatic behaviour decreased. Additionally, EF and WM improved, and walking became more regular. In Cunha et al. (2023), participants in the VR intervention group (13 adults with ADHD symptoms as assessed by Diagnostic Interview for ADHD in adults DIVA 2.0; Kooij and Francken 2007) showed improvements in processing speed after VR training, with a significant group x time interaction.

Theoretically, results from NMs and findings from this study support a dimensional view in which overlapping surface measures of attention, fluency and PWM can arise from different cognitive organisations. Although PWM bridges attention and fluency, the role of attention in ADHD remains preserved. Thus, ADHD assessments may benefit from separating measure overlap to real‐time attentional performance and under sustained attention form, whereas fluency variations and PWM differences alone should not imply a stuttering‐like architecture. Although PWM assessments remain valuable to characterise the broader ADHD profile (Howell et al. 2017; Kazazi and Howell 2026), interventions should target attentional regulation under demands that these differences emerge rather than assuming similar differences between PWADHD and PWS and across all attention forms.

### 4.3 Limitations and Constraints on Generality

Findings in the present study are most directly applicable to (1) adults who speak conversational or fluent English; (2) are classified as neurotypical participants or having self‐reported ADHD symptoms; (3) are assessed in the ASRS, SSI‐3 and UNWR; and (4) are tested on the 10 VR attention tasks described here. We anticipate group differences to be observed in the CPT/TFT task (Task 2) in comparable samples recruited through university participant pools and online ADHD forums assessed in similar VR paradigms under similar hardware and task constraints. We do not assume generalisability to children or adolescents, to clinically diagnosed or medicated ADHD samples, to samples with formal screening for co‐occurring neurodevelopmental conditions or to non‐English‐speaking and culturally diverse cohorts. We also do not generalise to other attention measures not evaluated in the present study or to similar paradigms that utilise other technology platforms. Additionally, all tasks were implemented in Unity and HTC Vive VR headset with the built‐in Pupil labs eye tracker to collect behavioural data (this part) and eye‐tracking data (Part II). EEG data were recorded in all 10 VR tasks with a Looxid Link EEG dry‐electrode mask placed on the VR headset, but spectral and raw analyses are not reported. The forehead sensors were affected by blinks and movement, and data processing was not feasible. Direct replications should therefore preserve the same software engine, VR headset and eye‐tracking device. Replications of task 10 do not require the EEG mask used in the present study, which is no longer sold due to incompatibility with newer headsets. Replications require an equivalent real‐time threshold, which could be achieved from another BCI, built‐in sensor or a rule based on the head/gaze direction. We expect similar implemented immersive scenarios to retain the same attentional demands and ecological validity, but we do not claim that the attention forms assessed here will produce the same effects.

There are several other: First, although the sample was relatively balanced, Task 1 had a reduced number of participants due to technical recording issues. Sensitivity analyses indicated that the study was adequately powered for the large effects but limited to smaller effects, and the findings are interpreted as exploratory. Second, PWADHD were assigned to this group based on self‐reported ADHD rather than structured clinical assessments. Although LMM analysis showed that ASRS, SSI‐3 and UNWR scores in the present sample did not differ significantly from an independent clinically diagnosed ADHD sample assessed with the same measures, the findings are applicable to profiles with ADHD traits rather than with an ADHD diagnosis. In addition, ADHD presentations (inattentive, hyperactive–impulsive, combined) and symptom severity were not included in the current analysis as factors to explain differences in performance, limiting conclusions on differences between subtypes. Third, selection effects may have occurred due to different ways of recruiting participants. Fourth, gender was imbalanced in PWADHD (21 females, 13 males) and neurotypical participants (11 females, 19 males). Although such differences did not differ significantly between groups and group differences remained significant after controlling for age and gender, this imbalance may still limit generality given gender differences in ADHD. Fifth, correction for multiple testing was not conducted across tasks because the primary aim of the study was to explore attention differences between groups across forms of attention. The interpretation was not only based on *p*‐values but also on effect sizes, sensitivity power analyses and logistic regression classification. However, uncorrected Type I error risk remains. Sixth, task durations were intentionally kept short to minimise effects of fatigue and cognitive overload since all 10 VR tasks were completed within a single session. However, PWADHD typically exhibit differences in tasks that require sustained attention over prolonged periods. Hence, the absence of differences may reflect session structure and duration of the task. Tasks were counterbalanced to reduce order effects, but familiarity with VR, differences in comfort with head‐mounted displays and individual interaction styles may have also influenced performance. Although participants reported presence and realism and were familiarised with the environment before testing, individual differences cannot be excluded. Seventh, the designed VR scenarios are consistent with the verisimilitude approach to ecological validity (perceived resemblance to real‐life situations) instead of veridicality, which assesses whether performance predicts real‐world functioning (Chaytor and Schmitter‐Edgecombe 2003). Hence, the degree of how the present findings relate to everyday attentional difficulties remains to be further studied. Additionally, since participants knew that they were being assessed, results may reflect their maximal performance rather than their typical everyday executive performance (Toplak et al. 2013). Eighth, none of the included PWADHD reported medication use or co‐occurring diagnoses. Performance in tasks, results from background measures and VR interaction style may differ across clinical status, medication, co‐occurrence and socio‐linguistic background. Hence, the present findings should be considered as specific to this sample rather than representative of all ADHD populations. Finally, the present findings are based on group‐level analysis. Individual heterogeneity in attentional organisation may not be captured by between‐group comparisons, limiting the extent to which these findings can be generalised to the individual level.

### 4.4 Future Directions

Future studies should aim to extend current findings in several ways. First, studies can test whether the present findings replicate in larger sample sizes with balanced gender samples and in adults with a clinical diagnosis of ADHD. Second, future studies should evaluate (1) ADHD presentation, (2) symptom severity, (3) medication status and (4) co‐occurring neurodevelopmental conditions in sufficiently larger samples to clarify whether differences observed here generalise to clinical populations. Third, future analyses should correct for multiple comparisons and confirm findings across all forms of attention in longer tasks. Fourth, veridicality can also be examined by assessing whether performance in VR tasks relates to everyday functioning through self‐report ratings. Fifth, future work should model VR behavioural measures together with background measures of attention, fluency and PWM in sufficiently larger samples in neurotypical participants, PWADHD and PWS. A case can be made for including dual‐trait subgroups to model jointly real‐time attentional performance and organisation from background measures. Sixth, item‐level PWM metrics, additional components of SSI‐3 such as percentage of stuttered syllables (%SS), as well as indexing separately measures of ASRS (attention and hyperactivity/impulsivity) can be modelled to clarify whether differences in sustained attention tasks relate more to attentional regulation, inhibitory control, phonological storage or broader EF demands. Seventh, examining individual heterogeneity in larger and clinical samples may help determine whether results observed apply across individuals or reflect distinct subgroups. Finally, studies should incorporate a multimodal approach to assessing ADHD by combining behavioural data with oculomotor measures (explored in Part II), EEG, as well as actigraphy to assess whether the results observed here may improve classification. Concurrent EEG measures are desirable. EEG systems should preferably be designed to fit the VR headset and allow for research‐grade cleaning of raw data. Alternatively, a standard EEG system may be used, provided that the whole system remains light and comfortable for the participant.

### 4.5 Conclusions

In conclusion, this study assessed several forms of attention within the same VR framework and provided a comprehensive evaluation of attentional differences in PWADHD. Findings indicated that PWADHD showed differences predominantly in sustained attention tasks with infrequent targets. VR‐based assessment offers a promising approach for assessing form‐specific attentional differences and addressing inconsistencies in the traditional paradigms in the ADHD literature, provided that findings are interpreted within the constraints of the sample, measures, tasks and technology discussed here.

## Author Contributions

**Fjorda Kazazi:** software, data curation, investigation, formal analysis, methodology, visualization, writing – original draft, writing – review and editing, project administration, resources, conceptualization, validation. **Peter Howell:** conceptualization, supervision, writing – review and editing, resources, methodology.

## Ethics Statement

The study was approved by University College London (UCL) Research Ethics Committee (6252/002). Participants read and signed an information sheet and a consent form prior to conducting the study.

## Conflicts of Interest

The authors declare no conflicts of interest.

## Data Availability

The datasets generated and analysed for this study can be found in the paper's Open Science Framework Page: [https://osf.io/jwzan/overview?view_only=325c1cab55cc49d284b1d9b3ffb797a3] (this is a reviewers' only version; the repository will be made public upon acceptance).

## Endnotes

^1^
WHO stands for the World Health Organisation. DSM‐5 is the Diagnostic and Statistical Manual of Mental Disorders, Fifth Edition. The ASRS used in this study is the six‐item screening scale updated for DSM‐5 criteria.

^2^
Unity Game Engine: https://unity.com/.

^3^
Unity Asset Store: https://assetstore.unity.com/.

^4^
DAZ3D to create realistic human avatars: https://www.daz3d.com/.

^5^
Freesound for downloading sounds: https://freesound.org/.

^6^
IBM Watson text‐to‐speech: https://www.ibm.com/.

^7^
HTC Vive Pro: https://www.vive.com/uk/product/vive‐pro/.

^8^
Pupil labs eye‐tracking technology: https://docs.pupil‐labs.com/core/vr‐ar/vive/.

^9^
Alienware m15 Ryzen Edition R7 laptop: https://www.dell.com/en‐uk/shop/gaming‐laptops‐pcs‐and‐accessories/alienware‐m15‐gaming‐laptop/spd/alienware‐m15‐r7‐gaming‐laptop.
